# Transcriptional Rearrangements Associated with Thermal Stress and Preadaptation in Baikal Whitefish (*Coregonus baicalensis*)

**DOI:** 10.3390/ani14213077

**Published:** 2024-10-25

**Authors:** Yulia P. Sapozhnikova, Anastasiya G. Koroleva, Tuyana V. Sidorova, Sergey A. Potapov, Alexander A. Epifantsev, Evgenia A. Vakhteeva, Lyubov I. Tolstikova, Olga Yu. Glyzina, Vera M. Yakhnenko, Valeria M. Cherezova, Lyubov V. Sukhanova

**Affiliations:** Limnological Institute Siberian Branch of the Russian Academy of Sciences, 3 Ulan-Batorskaya, Irkutsk 664033, Russia; tuyana_be@mail.ru (T.V.S.); poet1988@list.ru (S.A.P.); epifantsevalexander@yandex.ru (A.A.E.); vakhteevaevgenia@mail.ru (E.A.V.); lubow.tur@yandex.ru (L.I.T.); glyzina@lin.irk.ru (O.Y.G.); vera@lin.irk.ru (V.M.Y.); lcherezova12@yandex.ru (V.M.C.); lsukhanova@yandex.ru (L.V.S.)

**Keywords:** differential gene expression, heat shock proteins, HSP, HSR, molecular chaperones, transcriptome, acclimation, thermal adaptation, immune response, thermal stress, whitefish

## Abstract

In this work, we describe the transcriptional profiles of preadapted and non-adapted one-month-old juvenile Baikal whitefish after heat shock exposure. Preadapted fish were exposed to a repeated thermal rise of 6 °C above the control temperature every three days throughout their embryonic development. One month after hatching, preadapted and non-adapted larvae were either kept at control temperatures (12 °C) or exposed to an acute thermal stress (TS) of 12 °C above the control temperature. In response to this acute stress, an increase in HSP gene expression (*HSP-30*, *HSP-40*, *HSP-47*, *HSP-70*, and *HSP-90*) and *TRIM16* was detected, independent of preadaptation. The expression levels of genes responsible for the response to oxygen levels, growth factors and the immune response, *HBA*, *HBB*, *Myosin VI*, *Myosin VII*, *MHC*, *Plumieribetin*, *TnI*, *CYP450*, and *LDB3* were higher in individuals that had previously undergone adaptation. Genes responsible for the regulation of metabolism, *MtCK*, *aFGF*, *ARF*, *CRYGB*, and *D-DT*, however, increased their activity in non-adapted individuals. This information on transcriptional profiles will contribute to further understanding of the mechanisms of adaptation of whitefish to their environment.

## Simple Summary

In this work, we tested the hypothesis that thermal preadaptation benefits Baikal whitefish juveniles. Our treatments were designed to mimic temperature fluctuations near industrial thermal effluent areas or during the rearing of fish in aquaculture and to clarify the preventive role of the preadaptation of whitefish eggs on the subsequent stress to their larvae. We investigated the effects of egg preadaptation and the subsequent higher thermal stress on transcriptional rearrangements in the larvae of thermally sensitive Baikal whitefish (*Coregonus baicalensis*), a keystone species that is promising for aquaculture in the Baikal region.

## 1. Introduction

Unfavorable husbandry conditions can cause aquaculture species to suffer from temperature stress, regardless of climate change [1]. The heat shock response (HSR) is an evolutionarily conserved cellular stress response found in all taxa, from bacteria to humans [2], and involves an increase in the levels of heat shock proteins (HSPs). In the folding, repair, and transport processes of other proteins, HSPs serve as molecular chaperones [2].

Although most of our understanding of stress biology stems from research on a limited number of model systems, fish represent perfect organisms to study HSR regulation [3]. Fish are ectothermic vertebrates that live in an aquatic environment with high thermal conductivity. Therefore, daily and seasonal temperature fluctuations have a significant impact throughout their lifetime [3]. To study the short- and long-term consequences of temperature stress in aquatic animals, fish provide useful models. According to Kelly et al. [4], fish have also become crucial models for developmental biology due to their external fertilization and the large, manipulable eggs and embryos of many species. It is also possible to study the modulation of the HSR in fish throughout their life cycle [3].

Environmental conditions can alter the properties of the HSR and its induction temperature in fish, just as in other vertebrates [5,6]. Several studies have reported that induction temperature can increase with acclimatization, latitudinal thermal gradients, and tide temperatures [7,8,9]. Taken together, these studies indicate that thermal history and species plasticity are linked and that the abilities of the HSR induction threshold could change in response to thermal history [7,8]. Nevertheless, most of these studies have concentrated on long-term temperature changes in the natural environment and have used adult fish or juveniles as models. While data on the embryos of different fish species are more limited, they have also been shown to develop an HSR after exposure to thermal stress, including the lake whitefish *Coregonus clupeaformis* [7,10], the zebrafish *Danio rerio* [11], the Atlantic salmon *Salmo salar* [12], and the Japanese medaka *Oryzias latipes* [13]. Many aspects of fish development, such as rate and timing, size at hatching, survival, the number and extent of abnormalities, and metabolic and cellular processes, have been shown to be influenced by incubation temperatures in aquaculture [10]. Moreover, early hatching was caused by daily fluctuations at an average daily temperature of 1–5 °C above ambient levels [7]. According to Thome et al. [14], in situ incubation experiments revealed that locations affected by thermal effluents from power plants experienced greater temperature fluctuations and had higher average daily temperatures than reference locations. These locations also yielded larger embryos with smaller yolk sacs, indicating accelerated growth. Nevertheless, there is not enough thorough research on how temperature fluctuations during the embryonic period affect different biological systems or the effects of the increased stress that follows preadaptation.

To date, the consequences of repeated exposure to stressors, such as those that can occur during the rearing of fish in aquaculture or through industrial thermal effluents, have not yet been investigated. In this context, it is even more interesting to study the influence of thermal preadaptation on the subsequent heat shock as a promising solution for the development of stress-resistant aquacultures [9]. For this work, we investigated the effects of thermal preadaptation on the Baikal whitefish, *Coregonus baicalensis* (Dybowski, 1874), a socio-economically important cool-water species endemic to Lake Baikal in the Russian Federation [15]. Thermal preadaptation through a temperature rise of 6 °C above the control (up to 9 °C, which was optimal for a given age) every 3 days, followed by a decrease, was performed throughout the embryonic development of Baikal whitefish (from 30 days post-fertilization). We then studied the HSR in preadapted and non-adapted one-month-old larvae of Baikal whitefish at a temperature of 12 °C above the control.

In general, these studies revealed the relationship between the thermal history of a species and adaptive plasticity, as well as how the induction threshold of this adaptation may change in response to thermal preadaptation. Numerous genes are dysregulated by thermal stress, as shown in a comprehensive review of previous research [5,7,8,16,17]. However, due to the complex and poorly understood nature of coregonid thermoregulation, it is currently challenging to determine physiological status based on existing diagnostic marker genes. Examining the transcriptome profiles of preadapted and non-adapted fish is most promising in this regard. Transcriptional profiling provides more information, although this trend in and of itself is not new. In this case, the additional contribution made by transcriptomics is to show how normal developmental gene expression patterns change in tandem with the HSR.

## 2. Materials and Methods

### 2.1. Fertilization, Incubation, and Preadaptation of Eggs

At the end of December 2022, male and female specimens of native Baikal whitefish were collected at water temperatures up to 2–4 °C in the spawning migration areas of Baikal whitefish in Chivyrkuisky Bay (Lake Baikal, Republic of Buryatia, Kurbulik settlement, 53°42′14.3″ N 109°02′16.8″ E). All work related to artificial fertilization was carried out according to guidelines that were developed on the basis of many years of experience in setting up temporary sites for egg collection and storage, physiological and embryological studies, data describing whitefish spawning conditions, and the physiological and morphological markers of egg development [18]. The eggs were fertilized using the dry method. For this purpose, well-mixed sperm from 4–7 males, collected immediately before use, was carefully added to the eggs collected from 6 females (approx. 1.2 L). Approximately 4 cc of the sperm mixture was added to each kg of eggs stored in clean and dry containers. Immediately after adding the sperm, the propagation products were carefully mixed with a bird feather to distribute the sperm evenly over the eggs. Then, a small amount of water was added (0.1–0.2 L per 1 kg of eggs), and the resulting mixture was again thoroughly mixed with water. The mixture of reproductive products and water was kept for at least 5–7 min. Successful fertilization of the eggs was assessed by the formation of a specific foam film on the water surface. After fertilization, the eggs were washed several times for 1 h and then kept until they were completely swollen. After the above procedures, the eggs were transported to the incubation site in a humid environment at a temperature of 0.5–4 °C.

The fertilized eggs were incubated in a system of 0.5-L smaller homemade copies of a Weiss apparatus (100–200 mL of eggs per apparatus) with aerated flowing Baikal water. Two types of Weiss apparatus were used to incubate the fertilized eggs: (1) the control apparatus, which incubated the eggs at a constant temperature of 3–3.5 °C; (2) the experimental apparatus, which started egg preadaptation at 30 days after fertilization (twice a week, the temperature was raised by 6 °C compared to the control and then lowered to the initial values). The temperature in the experimental apparatus increased by 6 °C over the period of 1 h, remained at 9 °C for 1 h, and then dropped to the initial level for another hour. The training lasted a total of 3 months. At this age, the physiological upper limit of the temperature norm for *C. baicalensis* is 7–14 °C [18], thus, these temperature fluctuations are environmentally realistic.

### 2.2. Experimental Design in Larvae

The techniques for maintaining one-month-old larvae at +12 °C and subsequent thermal treatment after hatching in April 2023 were developed on the basis of procedures at the Experimental Freshwater Aquarium Complex for Baikal Hydrobionts at the Limnological Institute (LIN SB RAS).

The thermal experiment was designed to correspond to the developmental characteristics of whitefish. From the moment the eggs hatched, the water temperature in all aquariums was gradually increased from 3–3.5 degrees to 12 degrees for one-month-old juveniles, which is physiologically appropriate for their age and is typical of the natural conditions in whitefish habitats [19]. To assess the stress response of both preadapted and non-adapted individuals, acute stress was produced at a sublethal temperature of 24 °C [19] when the larvae reached 1 month of age. The ideal water temperature range for the growth of juvenile whitefish is 13–18 °C [19]. However, temperatures above 24–26 °C can be fatal for juvenile whitefish [19,20]. Such fluctuations often occur in aquaculture when the cooling systems are not working or when the fish are transported [21], which is bad for fish health and survival. In addition, whitefish are known to be released into rivers while still in the larval stage in many fish farms [22]. However, despite high flexibility at this age, the larvae are non-adapted and may still experience stress.

**Figure 1:**
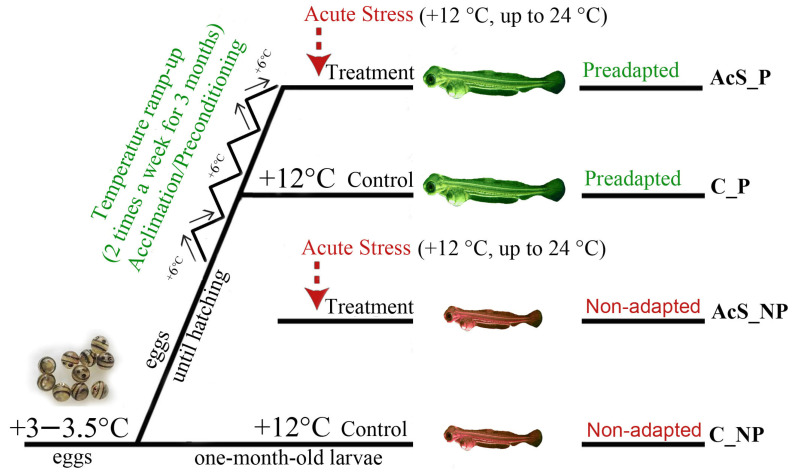
The temperature experiment on one-month-old Baikal whitefish larvae. The water temperature in the “acute stress for two hours” treatment aquariums was raised up to 24 °C (preadapted treatment (AcS_P) and non-adapted treatment (AcS_NP)), while the temperature in the “control” aquariums remained constant at 12 °C throughout the experiment (preadapted control (C_P) and non-adapted control (C_NP)).

The study examined both control individuals and individuals exposed to thermal stress (+12 °C relative to the control temperature of 12 °C, up to 24 °C, for two hours; room temperature during a simulated emergency shutdown of the refrigeration equipment). The listed control and treatment groups included both preadapted and non-adapted at the egg stage larvae (Figure 1).

### 2.3. Fish Larvae Collection and Ethical Standards

Preadapted and non-adapted whitefish larvae from the control and treatment aquariums were collected with a dip net 2 h after acute thermal exposure. The survival rate of pre-adapted individuals after acute stress was more than 2 times higher than that of non-adapted individuals (Table 1). Tricaine mesylate (MS222) was used to euthanize the whitefish from each aquarium, according to the 2020 AVMA Guidelines for Euthanasia of Animals. The samples were taken from the larvae that had been euthanized. The Ethics Committee of the Limnological Institute SB RAS accepted the experiments and the publication of the results in the press in accordance with Russian laws, standards, and guidelines on animal welfare (Protocol #2, 7 August 2024). A total of 53 one-month-old juveniles (12–16 fish per group) were analyzed (Table 1).

### 2.4. RNA Extraction, Library Construction and Sequencing

Total RNA was isolated from each sample (from the whole larvae) using TRIzol reagent (Thermo Fisher Scientific, Waltham, MA, USA). RNA quality was determined using an EzDrop1000 spectrophotometer (Blue-Ray Biotech, New Taipei City, Taiwan). To solve the problem of the limited number of replicates, 12–16 individuals per group were included in the analysis to compensate for individual differences. RNA samples from the same group were combined into mixtures to obtain four RNA pools.

Poly-T oligo-attached magnetic beads were used to separate the messenger RNA from the total RNA. After fragmentation, random hexamer primers were used to synthesize the first strand of cDNA. dTTP was used to synthesize the second strand of cDNA for a non-directional library. After end-repair, A-tailing, adaptor ligation, size selection, amplification, and purification, the non-directional library was prepared.

The library was analyzed with Qubit and real-time PCR for quantification and size distribution detection. The quantified libraries were pooled in equimolar amounts and sequenced on the Illumina NovaSeq 6000 high-throughput sequencer with a NovaSeq 6000 Reagent Kit v1.5 in 150 bp paired-end reads by Novogene Limited (Beijing, China). The Phred score was used to convert the error rate for each base, as shown in the equation Qphred = −10log10(e), where “e” represents the sequencing error rate and “Qphred” represents the base quality scores of the Illumina platform. To avoid poor-quality reads or reads with adapters, the raw reads were filtered with Trimmomatic v. 0.36 [23] to obtain clean reads.

### 2.5. De Novo Assembly

Transcriptome assembly was performed using a de novo method. The preprocessed reads were subjected to a de novo assembly procedure using the Trinity v.2.13.2. software package [24]. In this comprehensive assembly approach, default parameters were used to ensure a balanced representation of the transcriptome. Additionally, a predetermined minimum contig length of 300 nucleotides was set to filter out shorter fragments.

We then performed a quantitative assessment of the completeness of the data by contrasting our transcript set with a collection of highly conserved orthologs, characterized by their uniqueness. For this assessment, we used BUSCO (Benchmarking Universal Single-Copy Orthologs) version 5.5.0 [25], utilizing the eukaryota_odb10 database. Enumeration of complete BUSCOs (where the length is within two standard deviations of the average length for a given BUSCO), duplicate BUSCOs (where a single BUSCO is represented by multiple transcripts), fragmented BUSCOs (partially recovered BUSCOs) and missing BUSCOs (unrecovered orthologs) formed the basis of our analysis.

In order to obtain non-redundant transcript sets, a series of filtering steps were performed. Initially, TransDecoder v 5.7.1 [26] was used to identify putative coding regions within the compiled transcripts. Subsequently, a selection process was performed by maintaining the optimal open reading frame (ORF) for each transcript according to the TransDecoder pipeline, parameterized with the ‘--single_best_orf’ option. Transcripts containing ORFs with a length of less than 200 base pairs were subsequently excluded from further analysis. This rigorous filtering strategy ensured that a refined collection of non-redundant transcripts suitable for downstream analyses was generated.

Functional annotation of the obtained unigenes was performed using the DIAMOND tool [27] by querying the UniProtKB/SwissProt database (http://www.uniprot.org (accessed on 14 October 2024)) and additionally by the BLASTp tool in the following databases: KEGG (Kyoto Encyclopedia of Genes and Genomes, http://www.genome.jp/kegg (accessed on 14 October 2024)) and eggNOG (orthologous groups of genes, http://eggnogdb.embl.de (accessed on 14 October 2024)) [28]. Strict annotation criteria were implemented, specifying an e-value threshold of 0.00001 to ensure the selection of significantly homologous sequences.

### 2.6. Differential Gene Expression Analysis

Differential gene expression analysis was performed using the R package EdgeR v4.0.3. [29], Bowtie2 v2.3.5.1 [30], and Trinity v2.13.2 software components. EdgeR, a statistical framework, was used to analyze differential expression using an overdispersed Poisson model. The empirical Bayes method implemented in EdgeR was used to moderate the extent of overdispersion across genes [31].

Differentially expressed (DE) transcripts were filtered by applying a significance threshold set at *p* = 0.01, as well as a change in logarithmic ratio (logFC) = 1. However, to focus on the most highly expressed genes, we increased the threshold to obtain the top 20 genes. Strict filtering was achieved by applying a predetermined significance threshold of *p* = 0.001, as well as a minimum change in logarithmic ratio (logFC) = 2. The Pearson correlation method was used to calculate the distance matrix since the objective of clustering was to bring genes of a similar function together [32]. Heatmaps were built using the R programming language, using the package pheatmap v.1.0.12. Subsequently, the identified differentially expressed genes (DEGs) were annotated using the GenBank (v. 254) database. The annotation process was facilitated using the BLAST tool with a fixed parameter for an e-value of 0.001, ensuring the rigorous and precise assignment of functional information to the differentially expressed genes.

Functional prediction and the classification of DEGs were performed using the EggNOG mapper. An enrichment analysis of these DEGs was then performed with the KOBAS software v.3.0 [33], using genetic ontology (GO). The KOBAS software v.3.0 facilitated the statistical evaluation of gene enrichment within the Kyoto Encyclopedia of Genes and Genomes (KEGG) pathways. Genes that showed differential expression and with a corrected *p*-value below 0.05 were classified as significantly enriched, emphasizing their classification as differentially expressed genes of notable biological relevance.

### 2.7. Validation of Transcriptomic Data via qPCR

Total RNA from each sample (the whole larvae) was isolated with TRIzol reagent (Thermo Fisher Scientific, USA) and treated with RNase-Free DNase (Magen Biotechnology Co., Guangzhou, China) to remove any possible contamination with genomic DNA, used according to the manufacturer’s instructions. DNase inactivation was performed by adding 0.017 M EDTA (in its final concentration) and heating at 70 °C for 10 min, followed by sample purification using the Amplitech RNA-100 kit (Amplitech, Moscow, Russia). RNA quality and concentration were determined using an EzDrop1000 spectrophotometer (Bluy-Ray Biotech, Taiwan) and by agarose gel electrophoresis. The next step involved a reverse transcription reaction using random hexamer primers and the Reverta-L reagent kit for cDNA synthesis from an RNA template (AmpliSens, Moscow, Russia), used according to the manufacturer’s instructions.

Three significantly expressed genes, randomly selected, were used for qPCR analysis to verify the accuracy of the transcriptome data: *HSP-47*, *HSP-70*, and *HBB*. Glyceraldehyde-3-phosphate dehydrogenase (*GAPDH*) was chosen as a reference gene due to its sufficient investigation in previous studies [8,34], and its stability was tested with the Reference Gene Selection Tool in BioRad CFX96 software v.3.1 (Ln(1/AvgM) = 1.475). The primers for *GAPDH*, *HSP-47*, and *HSP-70* were taken from a previous study [8]. The *HBB* primers were designed in Primer-BLAST. All primers complied with the MIQE guidelines [35]. The specificity of the primers for the selected DEGs was checked with the transcriptome alignment to determine primer efficiency. The primer sequences are listed in Table 2.

A BioRad CFX96 Touch Real-Time PCR Detection System (BioRad, Hercules, CA, USA) was used to conduct qPCR analysis. The qPCR mixture contained 0.25 mM dNTPs, 0.2 U Snp-polymerase, 2.5 mM MgCl_2_ (Evrogen, Moscow, Russia), 1× Snp-buffer, 0.5-fold SYBR Green (Lumiprobe, Hunt Valley, MD, USA), 0.2–0.3 ng of DNA, and 0.5 pmol of each primer. The DNA polymerase was initiated at 95 °C for 3 min. The fragments of the target and reference genes were amplified simultaneously at a temperature of 60 °C for primer annealing. One cycle comprised three standard stages: 10 s at 95 °C, 15 s at 60 °C, and 15 s at 72 °C. The cycle was repeated 40 times. After each run, a melting curve analysis was performed to ensure that only a single amplification product was present.

The integrated software package v.3.1 of the BioRad amplifier was used to calculate the relative expression level of the target gene, based on the ΔΔCq method. The differences in expression levels between samples were estimated using a Kruskal–Wallis test (Statistica 10 software package). The results were considered statistically significant at *p* < 0.05.

## 3. Results

### 3.1. Transcriptome Sequencing and De Novo Assembly

**Figure A1:**
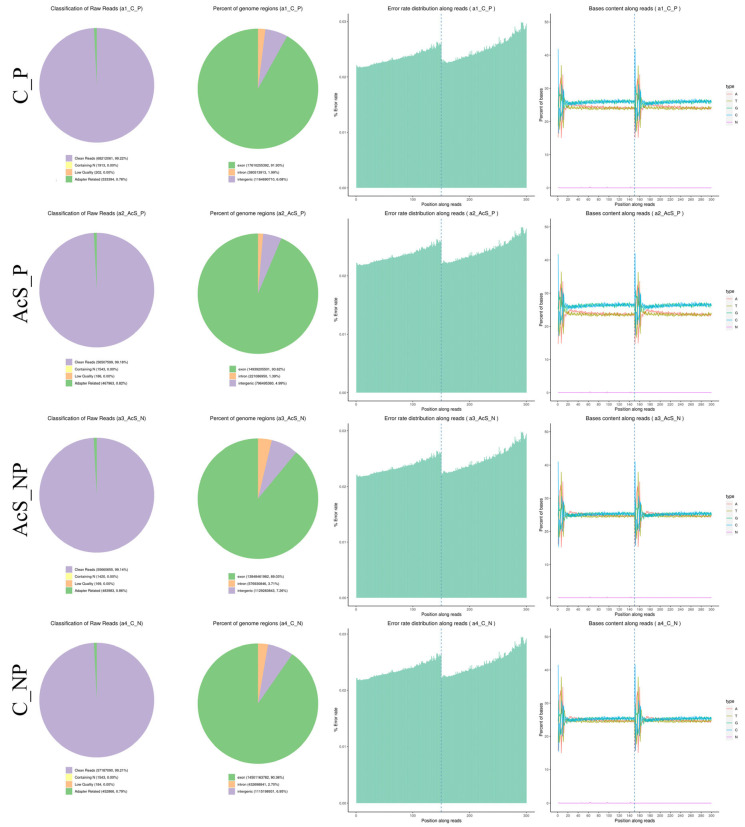
Sample sequencing, data filtering, sequencing reads in the genomic region, sequencing data error rate distribution, and GC content distribution. C_NP—non-adapted control, C_P—preadapted control, AcS_NP—non-adapted treatment (acute stress), and AcS_P—preadapted treatment (acute stress).

The sequencing data quality statistics are summarized in Table 3. The distribution of the sequencing reads (exons, introns, or intergenic regions) of all samples in the genomic region is shown in Figure A1. Throughout the sequencing process for the non-stranded library, the contents of G and C, A and T, which affect the measurement of gene expression, were equal.

The raw sequence data have been submitted to the Gene Expression Omnibus (NCBI-GEO) repository (accession number: GSE278874, https://www.ncbi.nlm.nih.gov/geo/query/acc.cgi?acc=GSE278874 (accessed on 15 October 2024)) and to the National Center for Biotechnology Information (NCBI) Sequence Read Archive (SRA) under BioProject ID #____________ (link will be available soon).

After the de novo assembly of the transcriptomes, a total of 727,333 contigs were identified. The minimum and maximum lengths were 180 and 16,494 bp, respectively, and the average length was 653.81 +/− 786.66 bp (see a detailed report in the Supplementary Materials—S1 and S2). The transcriptome assembly (TSA) can be viewed under the accession number ___________ (link will be available soon) or in the Gene Expression Omnibus (NCBI-GEO) repository (accession number: GSE278874, https://www.ncbi.nlm.nih.gov/geo/query/acc.cgi?acc=GSE278874 (accessed on 15 October 2024)).

**Figure 2:**
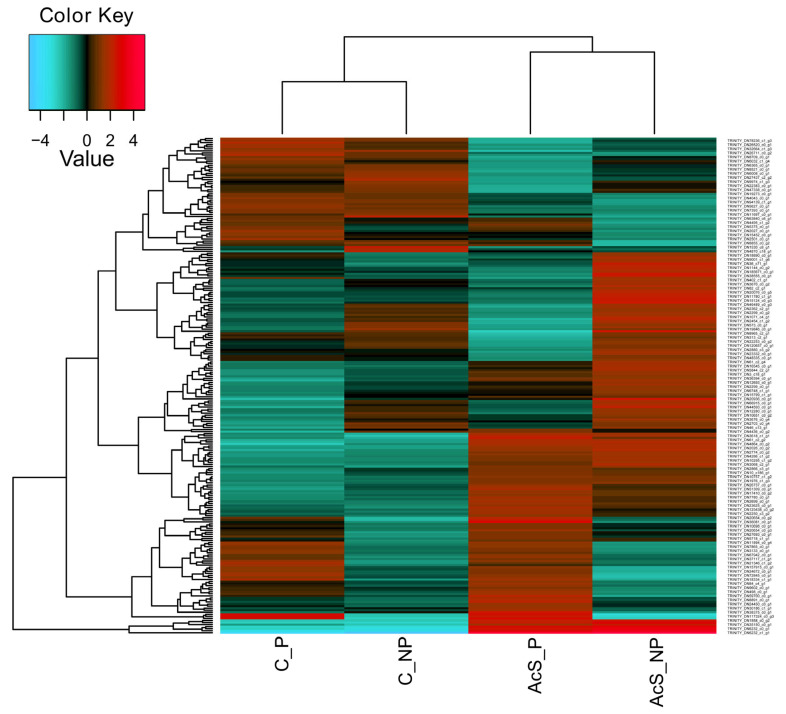
DEGs in Baikal whitefish after preadaptation and acute temperature rise. The heatmap shows the log fold change (logFC), *p* < 0.01. These are clustered according to similar expression patterns in the groups (Pearson correlation method). The complete counts can be found in the Supplementary Materials as S5 (logCPM) and S6 (logFC). Red indicates those genes with high expression levels, and green indicates those genes with low expression levels. C_NP—non-adapted control, C_P—preadapted control, AcS_NP—non-adapted treatment (acute stress), and AcS_P—preadapted treatment (acute stress).

Differential gene expression analysis was performed to detect variations in the transcriptional profile of Baikal whitefish larvae under different thermal conditions. The changes in gene expression in Baikal whitefish larvae in the control and experimental groups, which included both preadapted and non-adapted larvae at the egg stage, were compared. The total number of up-regulated and down-regulated DEGs with a threshold of significance, set at *p* = 0.01 (logFC = 1), was 242 (Figure 2, Table A1). The complete list of DEGs with detailed functions can be found in the Supplementary Materials as S3 (all DEGs without a given threshold) and S4 (DEGs with a significance threshold set at *p* = 0.01 (logFC = 1).

### 3.2. GO and KEGG Analysis of DEGs

**Figure 3:**
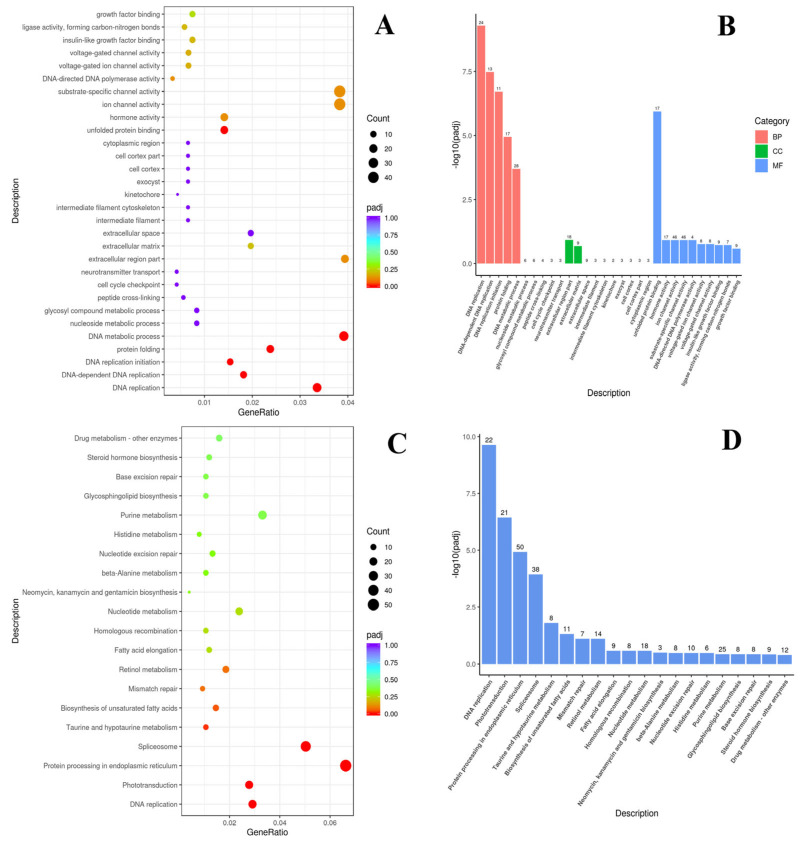
Comparison of the non-adapted groups of Baikal whitefish before and after acute stress (C_NP vs. AcS_NP): GO and KEGG enrichment analysis of DEGs. (**A**,**B**)—the most significant terms of GO databases, including biological processes (BP), cellular components (CC), and molecular function (MF); (**C**,**D**)—the top important terms for KEGG enrichment. C_NP—non-adapted control, AcS_NP—non-adapted treatment (acute stress).

GO and KEGG enrichment analyses were performed to gain an additional understanding of the biological relevance of the DEGs. The DEGs in the C_NP vs. AcS_NP groups were highly enriched in three functional categories, according to GO enrichment analysis: (1) biological process category (BP)—DNA replication, DNA-dependent DNA replication, DNA replication initiation, protein folding, DNA metabolic process, nucleoside metabolic process, and glycosyl compound metabolic process; (2) cellular components category (CC)—peptide cross-linking, cell cycle checkpoint, neurotransmitter transport, extracellular region part, extracellular matrix, extracellular space, intermediate filament, intermediate filament cytoskeleton, kinetochore, exocyst, cell cortex, cell cortex part, and cytoplasmic region; (3) molecular function category (MF)—unfolded protein binding, hormone activity, ion channel activity, substrate-specific channel activity, DNA-directed DNA polymerase activity, voltage-gated ion channel activity, voltage-gated channel activity, insulin-like growth factor binding, ligase activity, forming carbon-nitrogen bonds, and growth factor binding. The DEGs were mainly enriched in protein folding, unfolded protein binding, DNA replication initiation and DNA replication, DNA-dependent DNA replication, DNA metabolic processes, DNA-directed DNA polymerase activity, ion channel and substrate-specific channel activity, the extracellular region part, and hormone activity (Figure 3A,B).

According to the KEGG analysis, the DEGs were enriched in pathways such as drug metabolism and other enzymes; steroid hormone biosynthesis; base excision repair; glycosphingolipid biosynthesis; purine and histidine metabolism; nucleotide excision repair; beta-alanine metabolism; neomycin; kanamycin and gentamicin biosynthesis; nucleotide metabolism; homologous recombination; fatty acid elongation; retinol metabolism; mismatch repair; biosynthesis of unsaturated fatty acids; taurine and hypotaurine metabolism; spliceosome; protein processing in the endoplasmic reticulum; phototransduction; and DNA replication. Among them, protein processing in the endoplasmic reticulum, spliceosome processes, DNA replication, mismatch repair, phototransduction, retinol metabolism, biosynthesis of unsaturated fatty acids, and taurine and hypotaurine metabolism were significantly enriched. The number of enrichments in protein processing in the endoplasmic reticulum and spliceosome processes pathways showed the highest values (Figure 3C,D).

**Figure 4:**
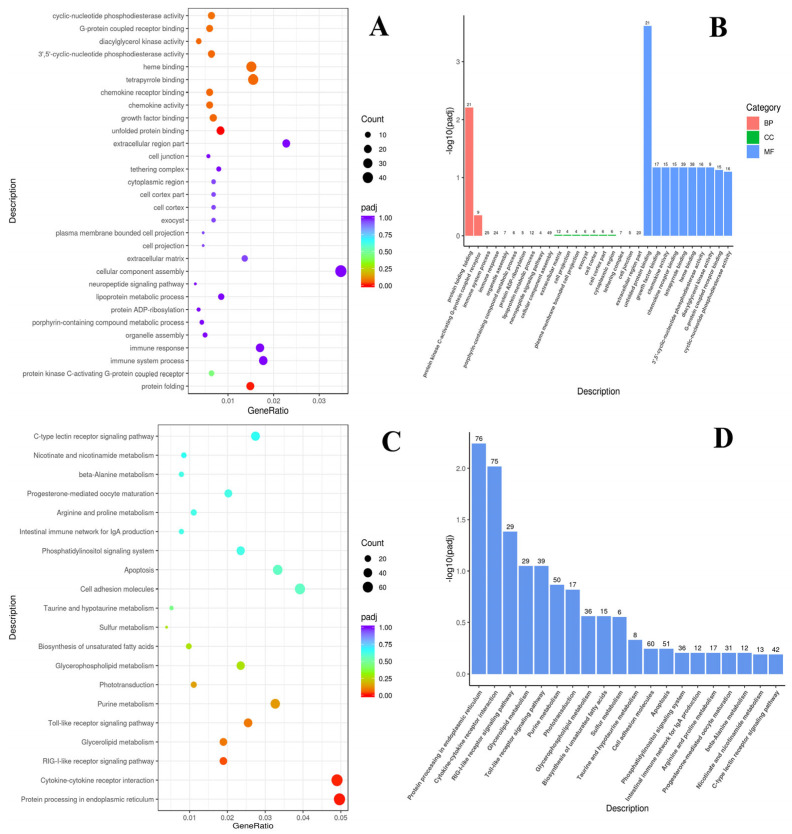
Comparison of the preadapted groups of Baikal whitefish before and after acute stress (C_P vs. AcS_P): GO and the KEGG enrichment analysis of DEGs. (**A**,**B**)—the most significant terms of GO databases, including biological processes (BP), cellular components (CC), and molecular function (MF); (**C**,**D**)—the top important terms for KEGG enrichment. C_P—preadapted control, AcS_P—preadapted treatment (acute stress).

The DEGs in the C_P vs. AcS_P groups were highly enriched in the following categories, according to the GO enrichment analysis: (1) biological process category (BP)—protein folding and protein kinase C-activating G-protein; (2) cellular components category (CC)—immune system process, immune response, organelle assembly, porphyrin-containing compound metabolic process, protein ADP-ribosylation, lipoprotein metabolic process, neuropeptide signaling pathway, cellular component assembly, extracellular matrix, cell projection, plasma membrane bounded cell projection, exocyst, cell cortex, cell cortex part, cytoplasmic region, tethering complex, cell junction, and extracellular region part; (3) molecular function category (MF)—unfolded protein binding, growth factor binding, chemokine activity, chemokine receptor binding, tetrapyrrole binding, heme binding, 3′,5′-cyclic-nucleotide phosphodiesterase activity, diacylglycerol kinase activity, G-protein coupled receptor binding, and cyclic-nucleotide phosphodiesterase activity. The DEGs were significantly enriched in terms of protein folding and unfolded protein binding, tetrapyrrole and heme binding, growth factor binding, chemokine activity, 3′,5′-cyclic-nucleotide phosphodiesterase activity, diacylglycerol kinase activity, G-protein coupled receptor binding, and cyclic-nucleotide phosphodiesterase activity (Figure 4A,B).

According to the KEGG analysis, the DEGs were enriched in pathways such as the C-type lectin receptor signaling pathway; nicotinate and nicotinamide metabolism; beta-alanine metabolism; progesterone-mediated oocyte maturation; arginine and proline metabolism; the intestinal immune network for IgA production; the phosphatidylinositol signaling system; apoptosis; cell adhesion molecules; taurine and hypotaurine metabolism; sulfur metabolism; the biosynthesis of unsaturated fatty acids; glycerophospholipid metabolism; phototransduction; purine metabolism; the toll-like receptor signaling pathway; glycerolipid metabolism; the RIG-l-like receptor signaling pathway; cytokine-cytokine receptor interaction; and protein processing in endoplasmic reticulum. It is notable that protein processing in the endoplasmic reticulum, cytokine–cytokine receptor interaction, RIG-l-like receptor signaling, the toll-like receptor signaling pathway, and glycerolipid metabolism pathways were significantly enriched, while the number of enrichments in protein processing in the endoplasmic reticulum and cytokine–cytokine receptor interaction pathways was the highest (Figure 4C,D).

**Figure A2:**
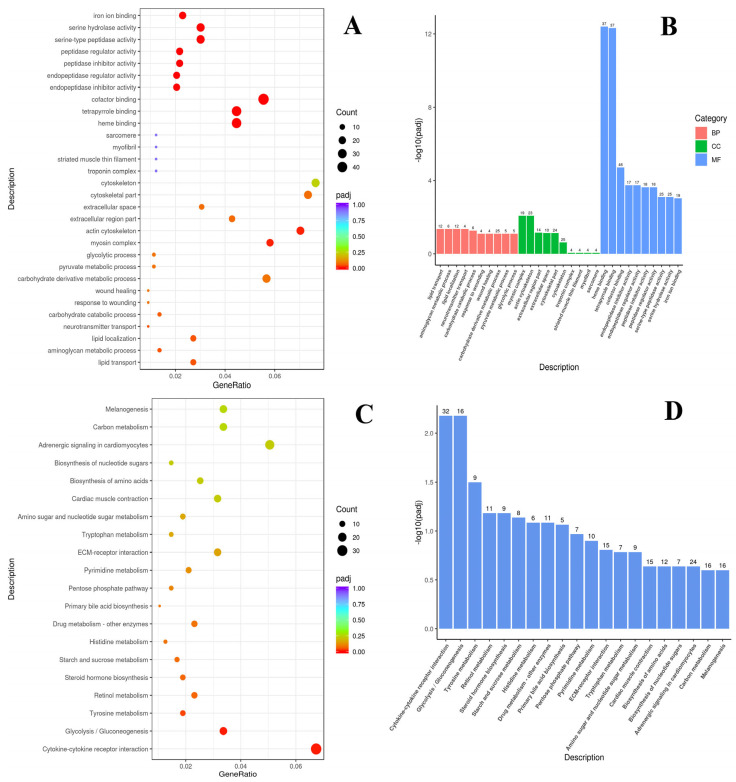
Comparison of the control preadapted and non-adapted groups of Baikal whitefish (C_P vs. C_NP): GO and KEGG enrichment analysis of DEGs. (**A**,**B**)—the most significant terms of GO databases, including biological processes (BP), cellular components (CC), and molecular function (MF); (**C**,**D**)—the most important terms for KEGG enrichment. C_NP—non-adapted control, C_P—preadapted control.

The DEGs in the C_P vs. C_NP groups were highly enriched in the following categories: (1) biological process category (BP)—lipid transport and localization, aminoglycan metabolic, carbohydrate catabolic and derivative metabolic processes, pyruvate metabolic and glycolytic processes, neurotransmitted transport, response to wounding, and wound healing; (2) cellular components category (CC)—myosin complex, actin cytoskeleton, extracellular region and cytoskeletal parts, extracellular space, troponin complex, striated muscle thin filament, myofibril, and sarcomere; (3) molecular function category (MF)—heme binding, tetrapyrrole binding, cofactor binding, endopeptidase inhibitor and regulator activities, peptidase inhibitor and regulator activities, serine-type peptidase and serine hydrolase activities, and iron ion binding. The DEGs were significantly enriched in almost all of the listed processes (Figure A2A,B).

According to the KEGG analysis, the DEGs were enriched in pathways such as cytokine-cytokine receptor interaction; glycolysis/gluconeogenesis; retinol and tyrosine metabolism; starch and sucrose metabolism; steroid hormone biosynthesis; histidine metabolism; drug metabolism and other enzymes; primary bile acid biosynthesis; melanogenesis; carbon metabolism; adrenergic signaling in cardiomyocytes; biosynthesis of nucleotide sugars and amino acids; cardiac muscle contraction; amino sugar and nucleotide sugar metabolism; tryptophan metabolism; ECM-receptor interaction; pyrimidine metabolism; and pentose phosphate. It is noteworthy that cytokine-cytokine receptor interaction, glycolysis/gluconeogenesis, tyrosine, histidine and retinol metabolism, steroid hormone biosynthesis, and starch and sucrose metabolism were significantly enriched, with the highest level of enrichment in the pathways for adrenergic signaling in cardiomyocytes and cytokine–cytokine receptor interaction (Figure A2C,D).

**Figure A3:**
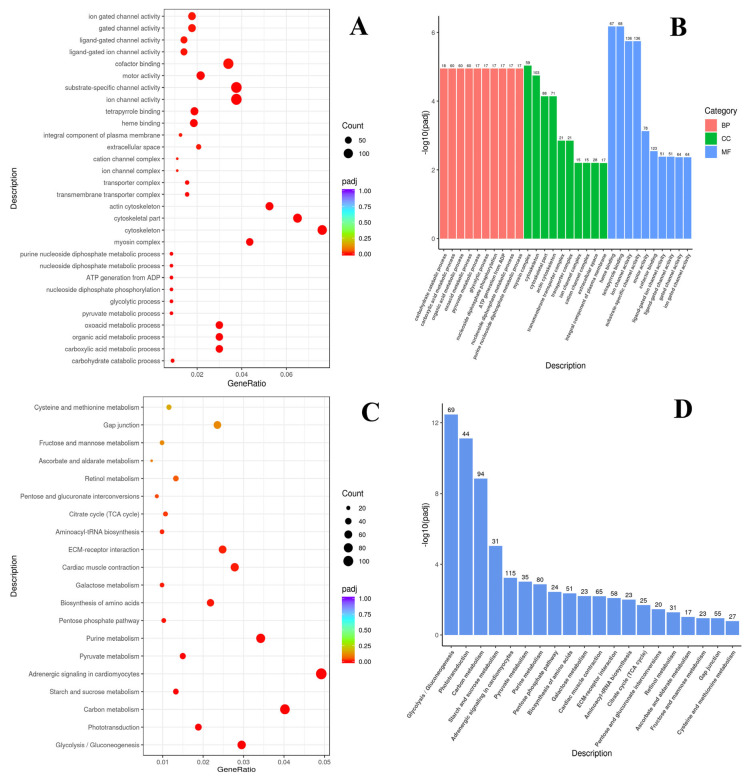
Comparison of the two groups exposed to acute thermal stress of Baikal whitefish with and without preadaptation (AcS_P vs. AcS_NP): GO and KEGG enrichment analysis of DEGs. (**A**,**B**)—the most significant terms of GO databases, including biological processes (BP), cellular components (CC), molecular function (MF); (**C**,**D**)—the top important terms for KEGG enrichment. AcS_NP—non-adapted treatment (acute stress), AcS_P—preadapted treatment (acute stress).

Finally, the DEGs in the AcS_P vs. AcS_NP groups were highly enriched in the following categories: (1) biological process category (BP): carbohydrate catabolic process, carboxylic acid metabolic process, organic acid metabolic process, oxoacid metabolic process, pyruvate metabolic process, glycolytic process, nucleoside diphosphate phosphorylation, ATP generation from ADP, nucleoside diphosphate metabolic process, and purine nucleoside diphosphate metabolic process; (2) cellular components category (CC)—myosin complex, cytoskeleton, cytoskeletal part, actin cytoskeleton, transmembrane transporter complex, transporter complex, ion channel complex, cation channel complex, extracellular space, and the integral component of plasma membrane; (3) molecular function category (MF)—heme binding, tetrapyrrole binding, ion channel activity, substrate-specific channel activity, motor activity, cofactor binding, ligand-gated ion channel activity, ligand-gated channel activity, gated channel activity, and ion gated channel activity. The DEGs were significantly enriched in all processes (Figure A3A,B).

According to the KEGG analysis, the DEGs were enriched in pathways such as cysteine and methionine metabolism; gap junction; fructose and mannose metabolism; ascorbate and aldarate metabolism; retinol metabolism; pentose and glucuronate interconversions; citrate cycle (TCA cycle); aminoacyl-tRNA biosynthesis; ECM-receptor interaction; cardiac muscle contraction; galactose metabolism; biosynthesis of amino acids; pentose phosphate pathway; purine and pyruvate metabolism; adrenergic signaling in cardiomyocytes; starch and sucrose metabolism; carbon metabolism; phototransduction; and glycolysis/gluconeogenesis. Among them, the levels of enrichment in adrenergic signaling in cardiomyocytes, in carbon and purine metabolism, and in the glycolysis/gluconeogenesis pathways were the highest (Figure A3C,D).

### 3.3. Analysis of the Top 20 DEGs

**Figure 5:**
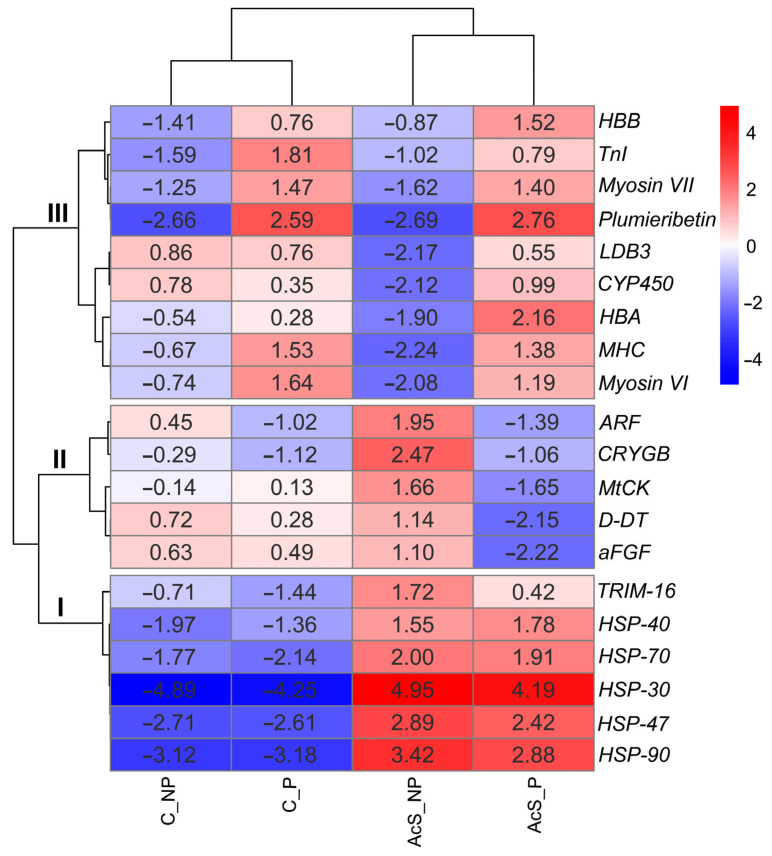
Top 20 DEGs in Baikal whitefish after preadaptation and acute temperature rise. The heatmap shows the log fold change (logFC), *p* < 0.001. These are clustered according to similar expression patterns in the groups (Pearson correlation method). Red indicates those genes with high expression levels (up-regulated DEGs), and blue indicates those genes with low expression levels (down-regulated DEGs). C_NP—non-adapted control, C_P—preadapted control, AcS_NP—non-adapted treatment (acute stress), AcS_P—preadapted treatment (acute stress).

To focus on the most highly expressed genes, we increased the threshold to *p* = 0.001 (logFC = 2), thus obtaining the top 20 DEGs that indicate transcriptional rearrangements in Baikal whitefish caused by mild heat stress during preadaptation in eggs and the subsequent acute stress in larvae. The expression patterns of the top 20 DEGs in all groups have been visualized in a heatmap (Figure 5).

In an experiment on the effects of thermal stress on the larvae of Baikal whitefish, clustering showed the division of differentially expressed genes into three groups, grouped according to similar expression patterns (Figure 5): (I) genes responsible for transmitting a signal that activates the stress response (*HSP-30* (*YRO1*), *HSP-40* (*DNAJ*), *HSP-47* (*SERPIN*), *HSP-70* (*HSPA*), *HSP-90* (*HSPC*), and Tripartite motif 16 (*TRIM16*); (II) genes responsible for the regulation of metabolism (Mitochondrial creatine kinase (*MtCK*), ADP-ribosylation factor (*ARF*), Acidic fibroblast growth factor (*aFGF*), D-dopachrome decarboxylase (*D-DT*), and ɣ-crystallin B (*CRYGB*)); (III) a group of genes responsible for oxygen levels, growth factors, and the immune response (Hemoglobin subunit beta (*HBB*), Fast myosin heavy chain (*MHC*), Mannose-specific lectin (*Plumieribetin*), Troponin-I fast skeletal muscle (*TnI*), Hemoglobin subunit alpha (*HBA*), *Myosin VI*, *Myosin VII*, Cytochromes P450 (*CYP450*), and LIM domain binding 3 (*LDB3*)). Genes in the first group, which mainly included HSPs, significantly increased their expression in response to acute thermal stress, regardless of preadaptation, while the expression of the genes in the third group was only higher in individuals that had previously undergone temperature adaptation at the egg stage. Genes responsible for the regulation of metabolism also differed between the groups studied; they showed increased activity in non-adapted individuals after acute thermal stress.

### 3.4. Validation of Transcriptomic Data via qPCR

**Figure 6:**
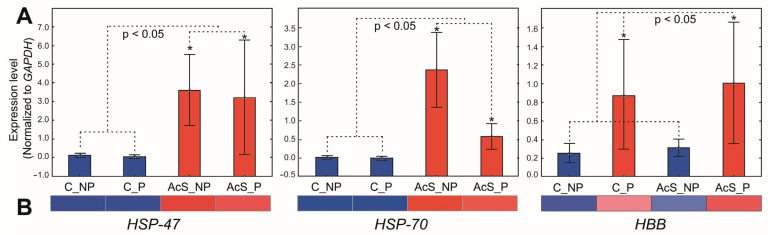
Comparison of the expression levels of DEGs detected by qPCR (**A**) and the data of gene-corresponding patterns from RNA-seq (**B**) in Baikal whitefish. Significant statistical differences between the experimental and control groups are indicated by an asterisk: * *p* < 0.05 (Kruskal–Wallis test). C_NP—non-adapted control, C_P—preadapted control, AcS_NP—non-adapted treatment (acute stress), AcS_P—preadapted treatment (acute stress).

The qPCR results (Figure 6A) showed a similar trend to the RNA-Seq results (Figure 5 and Figure 6B), indicating credible transcriptome data.

## 4. Discussion

### 4.1. Genes Responsible for Transmitting a Signal That Activates the Stress Response

In this work, the transcriptomic approach has allowed an investigation of stress-induced genes and revealed the complexity of expression patterns in response to thermal stress (AcS_P and AcS_NP groups, Figure 5) and preadaptation (C_P and AcS_P groups, Figure 5).

First, we found that exposure to thermal stress at 24 °C, regardless of preadaptation, significantly alters protein processing in the endoplasmic reticulum, protein folding, and the binding of unfolded proteins in Baikal whitefish (Figure 3 and Figure 4). This is confirmed by the significant enrichment of amino acid and carboxylic acid biosynthesis pathways, according to the GO and KEGG analyses (Figure 3 and Figure 4), and energy-consuming protein processing, which is related to an increase in HSPs (AcS_P and AcS_NP groups, Figure 5). This change is particularly reflected in the increased expression of the TRIM family protein, Tripartite motif 16 (*TRIM-16*), and HSPs, namely, *HSP-30* (*YRO1*), *HSP-40* (*DNAJ*), *HSP-47* (*SERPIN*), *HSP-70* (*HSPA*), *HSP-90* (*HSPC*) (Figure 5, Table A2), which is consistent with previous findings. In particular, certain fish species, including the lake whitefish *Coregonus clupeaformis* [7], the maraena whitefish *Coregonus maraena* [16], the rainbow trout *Oncorhynchus mykiss* [36], the Atlantic salmon *Salmo salar* [37], and the Hong Kong catfish *Clarias fuscus* [17] also show similar transcriptomic responses to comparable thermal challenges. Apparently, HSPs, also called chaperones, increased their expression independent of preadaptation, primarily to reduce the susceptibility of the experimental individuals to acute increases in temperature. This response was a kind of first line of defense to stabilize other cellular proteins involved in the stress response. Since most of the inducible HSP genes do not contain introns, the mRNA is rapidly translated into a nascent protein within minutes of exposure to a stressor [3]. Indeed, small heat shock proteins (sHSPs), ranging in size from 15 to 43 kDa, are widely recognized as a family of ATP-independent chaperones capable of trapping misfolded proteins through so-called “holdase” activity and preventing aggregation [38]. To then bind the unfolded or misfolded proteins and promote either refolding by a “foldase” activity or the proteolytic removal of the altered proteins, cooperation with ATP-dependent chaperones, such as *HSP-70* and *HSP-90*, is required [39].

*TRIM-16* and the chaperones *HSP-30*, *HSP-40*, *HSP-47*, *HSP-70*, and *HSP-90* were strongly up-regulated after acute stress in both experimental groups (AcS_P and AcS_NP groups, Figure 5). According to previous studies, *TRIM-16* controls the stress-induced biogenesis process, which strengthens the cellular antioxidant system to achieve cytoprotective benefits [40]. *TRIM-16* activates p62 and the genes involved in the ubiquitin pathway via NRF2 under conditions of oxidative and proteotoxic stress, leading to the ubiquitination of misfolded proteins and the formation of protein aggregates [40]. Therefore, according to Prajapati et al. [41], *TRIM-16* accelerates the stress-induced aggregate clearance process and protects cells from oxidative/proteotoxic stress-induced damage.

The function of HSPs is similar. They are also essential for reducing the negative consequences of stress by enabling the refolding of denatured proteins and, thus, preventing irreversible damage [42,43]. Furthermore, HSPs have been shown to be crucial for intercellular communication, DNA replication, signal transduction, cell differentiation, cellular senescence or immortalization, and the expression regulation of other genes; finally, they also support cell survival, possibly by averting apoptosis [17,42]. *HSP-70* and *HSP-90* have been extensively studied for their stress-responsive upregulation of expression and their critical function in cellular defense against many stressors [43,44]. In addition, the *HSP-40* family enhances the ATPase function of *HSP-70* through its co-chaperone activity, facilitating effective protein folding [45]. More detailed information can be found in Table A2.

In addition, we can also hypothesize a preliminary activation of HSPs during egg preadaptation (temperature ramp-up of +6 °C), which could have led to better survival and greater resilience of the preadapted individuals after thermal stress (Table 1). This is confirmed by the difference between preadapted and non-adapted individuals at the level of the following pathways (Figure A2 and Figure A3): glycolysis/gluconeogenesis, cytokine–cytokine receptor interaction, ATP generation from ADP, the purine nucleoside diphosphate metabolic process, heme binding, ion channel activity, substrate-specific channel activity, cofactor binding, adrenergic signaling in cardiomyocytes, enrichment in the adrenergic signaling in cardiomyocytes, carbon and purine metabolism, and others. In addition to inhibiting apoptosis, these alterations appear to have multiple cytoprotective effects on mitochondria, the sarcoplasmic reticulum, and cytoskeleton components [46], and also play an important role in maintaining enzymatic activity, insulin sensitivity, and glucose transport [47].

Thus, our experiments have shown that thermal stress in Baikal whitefish could affect the expression profile of genes that transmit a signal to trigger an adequate stress response, such as HSPs, and that these genes may be involved in mitigating the ecological risks associated with rising temperatures. This seems to indicate that the Baikal whitefish uses an adaptive mechanism to maintain proteostasis and provide protection against heat-related harm.

### 4.2. Genes Responsible for the Regulation of Metabolism

Our study has shown that high temperatures can lead to oxidative stress, but that preadaptation has a preventive effect in this regard. In particular, the activation of intracellular processes and the immune response after an acute increase in temperature was evident in preadapted individuals, according to the following pathways (GO and KEGG, Figure 4): the immune system process, immune response, the intestinal immune network for IgA production, organelle assembly, neuropeptide signaling pathway, cellular component assembly, growth factor binding, chemokine activity, diacylglycerol kinase activity, G-protein coupled receptor binding, cyclic-nucleotide phosphodiesterase activity, cytokine-cytokine receptor interaction, RIG-l-like receptor signaling, the toll-like receptor signaling pathway, and glycerolipid metabolism, while non-adapted individuals exhibited the processes associated with genomic stability, the induction of apoptosis, and DNA damage after an acute increase in temperature (GO and KEGG, Figure 3): DNA replication initiation and DNA replication, DNA-dependent DNA replication, DNA metabolic processes, ion channel and substrate-specific channel activity, extracellular region parts, and mismatch repair. In addition, a significant increase in the expression level of mitochondrial creatine kinase (*MtCK*) was observed in non-adapted individuals after acute temperature stress (AcS_NP group, Figure 5), while the expression of *MtCK* was significantly lower in preadapted individuals after thermal stress (AcS_P groups, Figure 5). Indeed, *MtCK* and other enzymes, such as acidic fibroblast growth factor (*aFGF*), ADP-ribosylation factor (*ARF*), D-dopachrome decarboxylase (*D-DT*), and ɣ-crystallin B (*CRYGB*) may serve as biomarkers for oxidative stress, as previous studies have shown (Table A3).

*MtCK* is a key regulator of cellular energy homeostasis and is located in the mitochondria of tissues with high energy demands [48,49]. This enzyme provides energy for numerous cellular functions in the brain and cardiac tissue, and especially in skeletal muscles, by catalyzing the conversion of creatine and adenosine triphosphate (ATP) to phosphocreatine (PCr) and adenosine diphosphate (ADP) [50]. Accordingly, an increase in *MtCK* denotes muscle damage due to stress or pathological processes [49]. Many conditions, including a rise in temperature, can cause the disruption of *MtCK* levels, meaning that they can serve as a sufficient diagnostic marker for acute stress [51]. At the same time, preadaptation may be a preventive measure for the increase in *MtCK* levels with a “memory/immune” effect seen in larvae subsequently exposed to acute stress (AcS_P group, Figure 5).

Similar expression patterns to *MtCK* were observed for acidic fibroblast growth factor (*aFGF*) and D-dopachrome decarboxylase (*D-DT*), where significantly decreased expression was observed as a “memory” effect in preadapted individuals following temperature stress (AcS_P group, Figure 5), and, in contrast, it increased in non-adapted individuals exposed to stress (AcS_NP group, Figure 5). The potent mitogen *aFGF* reduces demyelination by inhibiting oxidative stress damage and reducing the oxidative stress signaling cascades [52,53]. It acts by activating specific cell-surface receptors that trigger intracellular tyrosine phosphorylation cascades, but a number of findings also suggest that *aFGF* enters the cells and has an intracellular function [54].

Increased expression levels of *D-DT* were observed in non-adapted individuals exposed to thermal stress (AcS_NP group, Figure 5). Other animals exposed to thermal stress have previously shown a comparable rise in *D-DT* expression as an enhanced immunological and oxidative stress response [55]. As a recently identified cytokine that regulates both innate and adaptive immune responses, *D-DT* belongs to the macrophage migration inhibitory factor (MIF) protein superfamily [56]. The *D-DT* protein is present in most tissues and appears to play a crucial role in survival during acute stress. However, the neutralization of *D-DT* has been shown to significantly reduce inflammation [55,56], which was apparently observed in preadapted individuals after acute stress (AcS_P group, Figure 5). This could be explained by the modulation of cell migration through the activation of the MAP kinase cascade, which counteracts the immunosuppressive effect of glucocorticoids [56].

The protein ɣ-crystallin B (*CRYGB*) also showed the greatest activity in non-adapted individuals after acute thermal stress (AcS_NP group, Figure 5). This protein has previously been shown to play an essential role in modulating a number of cellular processes associated with stress recovery and survival, including apoptosis, cytoskeletal stability, and protein degradation [57]. In addition to being present in the lens, this protein has also been detected in the extracellular fluids of the heart, brain, skeletal muscles, and kidneys, where it plays a pleiotropic role in different cellular processes [58] and is associated with changes such as enhanced oxidative capacity and mitochondrial activity [59]. Since this protein lacks a signal sequence to be secreted via the typical secretory pathway, it might be released upon cell death or via the exosomes under certain stress conditions [60].

Based on the expression patterns in the experimental groups, ADP-ribosylation factor (*ARF*) stands out among the biomarkers mentioned above. Interestingly, *ARF* expression was significantly lower in both groups of individuals that underwent preadaptation (AcS_P and C_P groups, Figure 5) than in two non-adapted groups (AcS_NP and C_NP groups, Figure 5). The Ras-related GTPases, which make up the ADP-ribosylation factor (*ARF*) proteins, are thought to regulate membrane traffic and stress responses [61]. In addition, these proteins have recently been reported to be involved in innate immunity [62]. In a recent study, the knockout of *ARF* was found to increase tolerance to oxidative stress but also decrease susceptibility to osmotic stress and increase melanin formation [62]. Thus, the factor of adaptation was of primary importance and may indicate the presence of a specific immune response in preadapted groups. At the same time, the level of *ARF* expression was significantly higher in non-adapted individuals after acute stress (AcS_NP group, Figure 5) compared to all other groups. The results of this study suggest that *ARFs* may be crucial in determining how aquatic animals respond to thermal stress, opening a new way for identifying the mechanisms of resilience to unfavorable environments.

### 4.3. Genes Responsible for Oxygen Levels, Growth Factors, and Immune Response

The following genes increased their expression after preadaptation, regardless of thermal stress (C_P and AcS_P groups, Figure 5): Hemoglobin subunit alpha (*HBA*), Hemoglobin subunit beta (*HBB*), *Myosin VI*, *Myosin VII*, Fast myosin heavy chain (*MHC*), Mannose-specific lectin (*Plumieribetin*), and Troponin-I fast skeletal muscle (*TnI*). According to their annotation, these genes belong to the group responsible for oxygen transport, growth factors, and the immune response, and appear to respond to thermal preadaptation (Table A4). Indeed, it has been previously reported [7,8,9] that fish exposed to a mild stressor, which could be considered as preadaptation, undergo an activation phase in which the innate immune response is enhanced (the hormesis effect).

In fish and mammals, the hemoglobin that binds heme is a tetrameric protein consisting of two α and two ß chains [63]. Hemoglobin alpha (*HBA*) is involved in oxygen transport from the gills to the different peripheral tissues. Hemoglobin beta (*HBB*) regulates the innate immune responses that are mediated by RIG-I/MDA5 in a pleiotropic manner [63]. To adapt to the diversity of environments, both types are important. The effect of high temperatures on globins has recently been shown in other fish species [63,64]. For example, the expression of the *HBB* gene in the gills of the Japanese flounder *Paralichthys olivaceus* markedly decreased when the fish was exposed to thermal stress [63].

A similar expression pattern to that in hemoglobin was found for myosin, the most important protein in fish muscles that determines the quality of the muscle [65]. Myosin heavy chain-embryonic (*MHC-emb*) controls muscle fiber size, fiber number, and fiber type at the cell-autonomous level, while regulating myogenic progenitors and myoblast differentiation at the non-cell-autonomous level [66]. *MHC-emb* also mediates the effects on myogenic progenitors and myoblasts via secreted fibroblast growth factor (*FGF*) signaling [66], a change that was also shown in this work for preadapted individuals (see Section 4.2). Fish myosin is generally unstable, especially that of cold-water fish. Nevertheless, the contractile apparatus of fish that live in a specific thermal habitat has evolved to withstand that particular temperature range [65]. Furthermore, temperature changes have such a profound effect on the development of muscle tissue and myosin that they can cause phenotypic diversification, as seen in the case of the freshwater whitefish *Coregonus lavaretus* [67] and as discussed in more detail in Section 4.4 of the current work. In this context, it becomes clear how acclimatization during embryonic development can affect the subsequent growth of larvae, mass, size, and survival rate (Table 1).

*TnI* and *Plumieribetin* showed similar expression patterns after preadaptation, as described above (C_P and AcS_P groups, Figure 5). *TnI*, the inhibitory subunit of the troponin complex in the sarcomeric thin filament of striated muscle, plays an essential role in the calcium regulation of contraction and relaxation [68]. *TnI* in skeletal muscle has been suggested to be a sensitive and rapid fiber-specific marker of alterations in skeletal muscle and may change to adapt to new environmental conditions [68]. In addition, the data demonstrate that *TnI* expression may change in the long term due to thermal preadaptation [68,69].

*Plumieribetin* is a homotetramer and contains a high content of anti-parallel beta strands, similar to those in the mannose-binding B-lectins [70]. This protein has been associated with innate immunity and protection against microbial and metazoan parasites, due to its mannose-binding activity [71]. In particular, *Plumieribetin* weakens cell-collagen contacts, reduces cell spreading, and alters the actin cytoskeleton. Among the homologous fish lectins, only pufflectin, from the skin and intestine of the torafugu *Takifugu rubripes*, has been identified so far [72]. Obviously, *Plumieribetin*, with its activity in preadapted individuals of Baikal whitefish, adds a completely new function to this class of B-lectin.

Slightly different patterns were found for the Cytochrome P450 (*CYP450*) and LIM domain binding 3 (*LDB3*) genes. In particular, we found that non-adapted individuals showed the suppression of *CYP450* and *LDB3* expression at elevated temperatures (AcS_NP group, Figure 5). In the preadapted individuals, the expression levels of *CYP450* and *LDB3* were close to those of the control (C_NP group, Figure 5) regardless of stress load (C_P and AcS_P groups, Figure 5), which may indicate the presence of adaptive mechanisms at the transcriptional level that help Baikal whitefish to regulate metabolic processes, increase stress tolerance, and adapt to adversity.

Previous reports have also demonstrated that changing temperatures can induce or inhibit *CYP450* expression [73,74], which is consistent with the results of the present study. Over the past 20 years, much work has been done to characterize fish CYP genes [75] and to gain insight into the regulation of the *CYP450* gene by environmental stress [73]. The metabolism of many different compounds, including fatty acids, cytokines, bile acids, biogenic amines, steroid hormones, and prostaglandins, is significantly influenced by the *CYP450* enzyme [76]. CYP enzymes are, therefore, essential for the maintenance of homeostasis and metabolism in cells, and their alteration indicates pathological processes [73,74].

The second protein, which also shows interesting expression patterns in response to thermal stress (AcS_NP group, Figure 5), is LIM domain binding 3 (*LDB3*). *LDB3* is a striated muscle-specific alternatively spliced Z-band protein that is involved in the alignment and bundling of membrane proteins and the remodeling of the mechanosensory actin cytoskeleton and also interacts with other proteins during cytoskeletal assembly [77]. By binding to the mechanosensitive regions of the cytoskeletal proteins linked to actin and some HSPs, *LDB3* functions as a multivalent interaction hub that supports cellular mechanosensing [78,79].

According to previous studies, thermal stress in experimental fish led to their experiencing energy-consuming responses, such as an accelerated metabolism and a reduction in energy reserves [9]. These effects may have an indirect impact on the ability of fish to develop effective long-term stress defense mechanisms [80]. Nevertheless, according to the current results, preadaptation may stimulate the innate immune response, which primarily involves changes at the expression level for different genes, such as *HBA*, *HBB*, *Myosin VI*, *Myosin VII*, *MHC*, *TnI*, *Plumbieribetin*, *CYP450*, and *LDB3*, and which could increase the thermotolerance of individuals during repeated thermal stress.

### 4.4. Ecological Effect of Adaptive Changes at the Level of Gene Expression

The evidence to date, and in this paper, suggests that organisms can maintain their physiological efficiency in response to changing environmental conditions through thermal preadaptation, and that this preadaptation can make them more resistant to subsequent higher thermal stress [7,9,16]. Adaptation occurs by switching enzymes and other biochemical systems to different temperature optima, thereby altering tolerance ranges to the prevailing environmental conditions, extending the limits of thermopreference, and enabling survival and successful existence under unfavorable thermal conditions in the environment [9,81]. Nevertheless, it is important to note that adaptive changes at the level of gene expression may have ecological and population effects that are likely to lead to the emergence of new forms. In particular, the findings of previous studies demonstrate that the growth potential of the normal-sized and dwarf forms of the freshwater whitefish *C. lavaretus* is dependent on thermal experience during early (embryonic) life [67]. The data suggest that different thermal experiences during embryonic life have lasting effects on the muscle growth of this ecotype pair and contribute to the development of the dwarf form. Thus, the effects of temperature variation on myosin expression and muscle tissue development are significant enough that they could lead to phenotypic diversification. Immunolabeling tests for Pax7, H3P, and Mef2 provide evidence that the biological mechanisms behind the enhanced growth rates after cold incubation in both ecotypes are increased proliferation and reduced differentiation rates in muscle precursor cells [67].

The data obtained for the freshwater whitefish *C. lavaretus* correlates well with the temperature-dependent change in the growth characteristics of Baikal whitefish in the current work and with the expression of genes responsible for oxygen levels, growth factors, and immune response, such as *HBA*, *HBB*, *Myosin VI*, *Myosin VII*, *MHC*, *Plumieribetin*, *TnI*, and *LDB3*. *MtCK* is also actively involved in these processes through energy transmission [50]. Extensive studies have been carried out to clarify the relationship between ATP and HSPs [82,83]. In particular, ATP deficiency boosted the phosphorylation of HSPs in epithelial cells, causing them to migrate from the cytoskeleton to the cytoplasm and promoting actin polymerization. In other studies, thermal stress has been shown to increase the expression of HSPs and activate antioxidant defense systems, while simultaneously downregulating ATP synthase and ATPase activity [83]. Overall, it is likely that ATP regulates the phosphorylation of HSPs, which stabilizes the cytoskeleton and inhibits apoptosis.

These patterns that have been identified in whitefishes are very comparable to those reported for two populations of Atlantic salmon (*Salmo salar*) with different spawning habits [84]. The results obtained when comparing the effects of different rearing temperatures on the embryos of a large benthic and a dwarf benthic morph of the Arctic charr *Salvelinus alpinus* [85] are also generally comparable to the present results.

Recently, much evidence has emerged on the influence of methylation on the expression of genes implicated in the thermal response. In particular, a unique control mechanism for stress-induced mRNA translation is provided by the m6A “reader” YTHDF2, which preserves the 5′UTR methylation of stress-induced transcripts under stress conditions. This, in turn, initiates the translation of HSPs in a cap-independent manner [86].

The other epigenetic mechanism explaining the temperature-dependent flexibility of muscle growth has been postulated based on molecular data from the Senegalese sole *Solea senegalensis* [87]. Rearing temperature was found to alter the methylation of the promoter of myogenin, a significant transcription factor that regulates myogenic precursor cell development [87]. It may be speculated that similar epigenetic processes are the basis for the temperature dependence of the transcriptomic response in Baikal whitefish, but this speculation requires further investigation.

In addition to the obvious effects of artificial factors such as aquaculture rearing or industrial thermal effluents, the current data are unquestionably significant in the context of climate change, since the transcriptomic response would undoubtedly alter in response to rising water temperatures at fish-spawning areas. For instance, a typical HSR has been seen in corals in response to natural warming, which involves a shift from growth-targeted genes to stress response genes [43,88,89].

Taken together, these findings demonstrate the relationship between species’ thermal history and adaptive plasticity, along with the possibility of a modification in the induction threshold of this adaptation in response to thermal history. However, the pathway from gene expression to stress tolerance may be extremely complicated and is governed by a number of biological systems and processes, including epigenetic features such as DNA methylation or the involvement of mobile elements. The underlying genes involved in the HSR are more numerous, diverse, and variable than previously thought, especially when considering their epigenetic modifications, variations, and co-interactions in the growth processes and immune responses. Therefore, future research should include the integration of transcriptomics and epigenomics.

## 5. Conclusions

The study shows that thermal preadaptation at an early stage of development can increase the subsequent stress resistance of juvenile Baikal whitefish and improve the immune response. This is probably due to the influence of the hormesis effect, which can be used as a quantitative indicator of biological plasticity. By hormesis, we mean a primary mild stress that leads to adaptive effects and, as a result, could contribute to ecological shifts. In particular, there is an increased expression of the genes responsible for growth, cellular metabolism, and homeostasis, such as *HBA*, *HBB*, *Myosin VI*, *Myosin VII*, *MHC*, *TnI*, *Plumieribetin*, and *TnI.*

In contrast, in response to thermal stress and the accumulation of misfolded proteins, the HSR rapidly triggers the upregulation of sHSPs and then of ATP-dependent chaperones to bind the unfolded or misfolded proteins. Thermal stress accelerates the production of reactive oxygen species and increases mitochondrial respiration, which, in turn, causes the transcriptional upregulation of *MtCK*, *aFGF*, *D-DT*, *CRYGB*, and *ARF* to prevent oxidative damage.

In general, transcriptome responses include the upregulation of genes in response to distress in non-adapted individuals and the preadaptation of genes in anticipation of stress in preadapted individuals, suggesting that the Baikal whitefish may enhance its ability to eliminate reactive oxygen species by increasing the activity of specific enzymes under thermal stress. According to the research, transcriptome resilience, i.e., the recovery of gene expression patterns to a control level after a period of adaptation, is comparable to ecological resilience and can be used as a benchmark to predict how different species will respond to stress. Furthermore, based on our results, the genes *HSP-30*, *HSP-40*, *HSP-47*, *HSP-70*, *HSP-90*, *TRIM-16*, *LDB3*, *CYP450*, *CRYGB*, *MtCK*, *aFGF*, *ARF*, and *D-DT* can be considered the most promising genes for further studies on thermal stress and the prevention and management of stress in fish juveniles. The molecular mechanisms underlying the adaptations of cold-water fish species to high temperatures need to be further investigated, as climate change may also pose additional environmental challenges, even for aquacultured fish kept in cages. However, we would like to emphasize that future research should, at least, include the integration of transcriptomics and epigenomics and should examine the effects of thermal stress on the proteome.

## Figures and Tables

**Table 1:** Characteristics of Baikal whitefish used in the study.

Group	Average Length, TL, cm	Weight, g	Survival Rate, %	Specimens Number
C_NP	15.88 ± 1.09	0.01 ± 0.002	100	16
C_P	22.75 ± 1.04	0.04 ± 0.006	100	12
AcS_P	23.38 ± 1.69	0.05 ± 0.015	85	12
AcS_NP	13.31 ± 2.14	0.01 ± 0.004	42	13

**Table 2:** Quantitative real-time PCR (qPCR) primers.

Name	Primer Sequence (5′-3′)	Resource	Product Size (bp)
*GAPDH* F	CCG TCC GTC TGG AGA AGG C	[8]	183 bp
*GAPDH* R	GAA GTG GTC GTT CAG AGC AAT G		
*HSP-47* F	ATG GGC AAG ATG GAG GAG AG	[8]	98 bp
*HSP-47* R	TCA GAC CAA GTT CAC CAA GAG		
*HSP-70* F	TCA TTA CAG TCC CCG CCT AC	[8]	139 bp
*HSP-70* R	TCA CCT CAA AGA TCC CAT CC		
*HBB* F	ACT TCA CCG CTG ATG TCC AG	designed in Primer-BLAST	112 bp
*HBB* R	CTG ATG GTC GTC GGT CTC TG		

**Table 3:** Sequencing data quality statistics: a1—C_P—preadapted control; a2—AcS_P—preadapted treatment (acute stress); a3—AcS_NP—non-adapted treatment (acute stress); a4—C_NP—non-adapted control.

Sample	Raw_Bases	Raw_Reads	Clean_Reads	Error_Rate	Q20	Q30	GC_pct
a1 (C_P)	20.62 G	137,495,140	136,424,122	0.02	98.26	94.87	51.62
a2 (AcS_P)	17.09 G	113,954,582	113,015,198	0.02	98.41	95.25	52.54
a3 (AcS_NP)	16.84 G	112,292,454	111,321,310	0.02	98.29	94.91	50.34
a4 (C_NP)	17.29 G	115,283,366	114,374,180	0.02	98.28	94.94	50.49

## Data Availability

The raw sequence data have been submitted to the Gene Expression Omnibus (NCBI-GEO) repository (accession number: GSE278874, https://www.ncbi.nlm.nih.gov/geo/query/acc.cgi?acc=GSE278874 (accessed on 15 October 2024)) and to the National Center for Biotechnology Information (NCBI) Sequence Read Archive (SRA) under BioProject ID #____________ (link will be available soon).

## Supplementary Materials

The following supporting information can be downloaded at: https://www.mdpi.com/article/10.3390/ani14213077/s1, Koroleva et al. [90], S1 (Transcriptome assembly report), S2 (Trinity gene counts), S3 (Annotation of all DEGs without a given threshold), S4 (Annotation of DEGs with a significance threshold set at *p* = 0.01 (logFC = 1)), S5 (DEG counts, logCPM), S6 (DEG counts, logFC).

## Appendix A

**Table A1:** The number of up-regulated and down-regulated DEGs in each selected group: C_NP—non-adapted control; C_P—preadapted control; AcS_P—preadapted treatment (acute stress); AcS_NP—non-adapted treatment (acute stress). The complete counts can be found in the Supplementary Materials in S5 (logCPM) and S6 (logFC).

Group	Up-Regulated DEGs	Down-Regulated DEGs
C_NP	91	151
C_P	104	138
AcS_P	132	110
AcS_NP	147	95

**Table A2:** The detailed functions of the genes responsible for transmitting a signal that activates the stress response (group I is in accordance with Figure 5).

Name	Functions	References
*HSP-30*	Prevents the aggregation of unfolded proteins caused by heat or chemicals by assembling the damaged protein into aggresomes, which reduces the harmful effects of aggregated protein scattered throughout the cell	[91,92]
*HSP-40*	Chaperoning intermediate filament; involved in major animal diseases, including cancer, autoimmune diseases, and neurodegenerative disorders; amplifies the ATPase function of HSP-70, facilitating effective protein folding	[45,93]
*HSP-47*	Maturation of collagen	[94]
*HSP-70*	Assists in the folding of a large variety of proteins	[42,43,44]
*HSP-90*	Assists in folding; maintenance of cytosolic proteins	[43,44]
Tripartite motif 16 (*TRIM16*)	Controls turnover of protein aggregates by modulating NRF2, ubiquitin system, and autophagy; inhibits cell proliferation through cell cycle regulation and dynamic nuclear localization, but commonly depends on protein–protein interactions to fulfill their cellular functions	[40,41]

**Table A3:** The detailed functions of the genes responsible for the regulation of metabolism (group II is in accordance with Figure 5).

Name	Functions	References
ADP-ribosylation factor (*ARF*)	Controls the assembly of vesicle coat proteins under stressful conditions, which, in turn, affects membrane traffic; plays a significant role in the response to stress under threat of apoptosis; regulates actin cytoskeleton remodeling and the secretory and endocytic pathways	[61,62]
D-dopachrome decarboxylase (*D-DT*)	An essential survival or anti-apoptotic factor; pro-inflammatory cytokine; controls the innate and adaptive immune responses; a member of the macrophage migration inhibitory factor (MIF) protein superfamily	[55,56]
Mitochondrial creatine kinase (*MtCK*)	Found in the mitochondria of tissues with high energy demands; provides a temporal and spatial energy buffer to maintain cellular energy homeostasis; involved in the organism’s energy metabolism and immune response; responsible for the chemical reaction that ensures muscle contraction; indicates muscle damage due to physical exertion, injury, or pathological processes	[48,49,50,51,82,83]
Acidic fibroblast growth factor (*aFGF*)	Alters the activity and modulates of numerous cell types; an effective and broad-spectrum mitogen; a mitogen for tumor and endothelial cells	[52,53,54]
ɣ-crystallin B (*CRYGB*)	Participates in muscle physiopathology; connected to the phenomenon of adaptations; plays a crucial role in the regulation of multiple cellular processes linked to survival and stress recovery, such as protein degradation, cytoskeletal stabilization, and apoptosis	[57,58,59,60]

**Table A4:** The detailed functions of genes responsible for the oxygen levels, growth factors, and immune response (group III is in accordance with Figure 5).

Name	Functions	References
Fast myosin heavy chain (*MHC*)	Determines the phenotypic of muscle fibers by means of competitive interactions with myosin genes	[66]
Troponin-I fast skeletal muscle (*TnI*)	Plays a crucial role in the calcium regulation of contraction and relaxation; the inhibitory subunit of the troponin complex in the sarcomeric thin filament of striated muscle	[68,69]
*Myosin VI*	Multifunctional actin-based motor protein; involved in intracellular cargo movement, attaching tail-associated cargoes to actin; implicated in several cellular processes including endocytosis, exocytosis, the maintenance of Golgi morphology, and epithelial cell migration	[65,66,67]
*Myosin VII*	Works by exerting local tension, especially at the plasma membrane, and moving cargoes; connected to lysosomes and may be involved in lysosome trafficking	[65,66,67]
Hemoglobin subunit alpha (*HBA*)	Involved in the transfer of oxygen from the gills to the different peripheral tissues; crucial for environmental adaptation	[63,64]
Hemoglobin subunit beta (*HBB*)	Serves as a pleiotropic regulator of the innate immune responses mediated by RIG-I/MDA5; important for adaptation to a variety of environments	[63,64]
Mannose-specific lectin (*Plumieribetin*)	Integrin inhibiting effect; pathogen defense; linked to innate immunity and protection against microbial and metazoan parasites	[70,71,72]
Cytochromes P450 (*CYP450*)	Member of the hemoprotein superfamily; essential to cellular metabolism and homeostasis; plays a pivotal role in the detoxification of xenobiotics; engaged in the mono-oxygenation processes of a broad variety of endogenous and foreign chemicals; regulated by environmental stresses	[73,74,75,76]
LIM domain binding 3 (*LDB3*)	Known as ZASP; engaged in protein kinase A signaling; plays a significant role in mechanosensory actin cytoskeleton remodeling; interacts with actin in cardiac and skeletal muscles; stabilizes the sarcomere during contraction; co-activates α5β1 integrins along with the protein TLN1	[77,78,79]

## References

[1] Korytár T., Nipkow M., Altmann S., Goldammer T., Köllner B., Rebl A. (2016). Adverse husbandry of maraena whitefish directs the immune system to increase mobilization of myeloid cells and pro-inflammatory responses. Front. Immunol..

[2] Georgopoulos C., Welch W.J. (1993). Role of the major heat shock proteins as molecular chaperones. Annu. Rev. Cell Biol..

[3] Basu N., Todgham A.E., Ackerman P.A., Bibeau M.R., Nakano K., Schulte P.M., Iwama G.K. (2002). Heat shock protein genes and their functional significance in fish. Gene.

[4] Kelly P.D., Chu F., Woods I.G., Ngo-Hazelett P., Cardozo T., Huang H., Kimm F., Liao L., Yan Y., Zhou Y. (2000). Genetic linkage mapping of zebrafish genes and ESTs. Genome Res..

[5] Tomalty K.M., Meek M.H., Stephens M.R., Rincón G., Fangue N.A., May B.P., Baerwald M.R. (2015). Transcriptional response to acute thermal exposure in juvenile chinook salmon determined by RNAseq. G3 Genes Genomes Genet..

[6] Olsvik P.A., Vikeså V., Lie K.K., Hevrøy E.M. (2013). Transcriptional responses to temperature and low oxygen stress in Atlantic salmon studied with next-generation sequencing technology. BMC Genom..

[7] Sessions K.J., Whitehouse L.M., Manzon L.A., Boreham D.R., Somers C.M., Wilson J.Y., Manzon R.G. (2021). The heat shock response shows plasticity in embryonic lake whitefish (*Coregonus clupeaformis*) exposed to repeated thermal stress. J. Therm. Biol..

[8] Manzon L.A., Zak M.A., Agee M., Boreham D.R., Wilson J.Y., Somers C.M., Manzon R.G. (2022). Thermal acclimation alters both basal heat shock protein gene expression and the heat shock response in juvenile lake whitefish (*Coregonus clupeaformis*). J. Therm. Biol..

[9] Sapozhnikova Y.P., Koroleva A.G., Yakhnenko V.M., Volkova A.A., Avezova T.N., Glyzina O.Y., Sakirko M.V., Tolstikova L.I., Sukhanova L.V. (2023). Thermal Preconditioning Alters the Stability of Hump-Snout Whitefish (*Coregonus fluviatilis*) and Its Hybrid Form, Showing Potential for Aquaculture. Biology.

[10] Whitehouse L.M., McDougall C.S., Stefanovic D.I., Boreham D.R., Somers C.M., Wilson J.Y., Manzon R.G. (2017). Development of the embryonic heat shock response and the impact of repeated thermal stress in early stage lake whitefish (*Coregonus clupeaformis*) embryos. J. Therm. Biol..

[11] Krone P.H., Lele Z., Sass J.B. (1997). Heat shock genes and the heat shock response in zebrafish embryos. Biochem. Cell Biol..

[12] Takle H., Baeverfjord G., Lunde M., Kolstad K., Andersen Ø. (2005). The effect of heat and cold exposure on HSP70 expression and development of deformities during embryogenesis of Atlantic salmon (*Salmo salar*). Aquaculture.

[13] Werner I., Koger C.S., Hamm J.T., Hinton D.E. (2001). Ontogeny of the heat shock protein, hsp70 and hsp 60, response and developmental effects of heat shock in the teleost, medaka (*Oryzias latipes*). Environ. Sci..

[14] Thome C., Mitz C., Somers C.M., Manzon R.G., Boreham D.R., Wilson J.Y. (2016). Incubation of lake whitefish (*Coregonus clupeaformis*) embryos in cooling water discharge and the impacts of fluctuating thermal regimes on development. Can. J. Fish. Aquat. Sci..

[15] Bychenko O.S., Sukhanova L.V., Azhikina T.L., Skvortsov T.A., Belomestnykh T.V., Sverdlov E.D. (2014). Differences in brain transcriptomes of closely related Baikal coregonid species. Biomed. Res. Int..

[16] Rebl A., Verleih M., Nipkow M., Altmann S., Bochert R., Goldammer T. (2018). Gradual and acute temperature rise induces crossing endocrine, metabolic, and immunological pathways in Maraena whitefish (*Coregonus maraena*). Front. Genet..

[17] Liu Y., Tian C., Yang Z., Huang C., Jiao K., Yang L., Duan C., Zhang Z., Li G. (2024). Effects of chronic heat stress on growth, apoptosis, antioxidant enzymes, transcriptomic profiles, and immune-related genes of Hong Kong catfish (*Clarias fuscus*). Animals.

[18] Chernyaev Z.A. (2017). Reproduction of whitefish. Ecological and Physiological Features of Reproduction and Development.

[19] Golovanov V.K. (2013). Ecological and physiological optimum temperature and upper temperature limits of coregonid fish life. Biology, Biotechnology of Breeding and the State of Stocks of Whitefish.

[20] Stewart T.R., Vinson M.R., Stockwell J.D. (2022). Effects of warming winter embryo incubation temperatures on larval cisco (*Coregonus artedi*) survival, growth, and critical thermal maximum. J. Great Lakes Res..

[21] Mugwanya M., Dawood M.A.O., Kimera F., Sewilam H. (2022). Anthropogenic temperature fluctuations and their effect on aquaculture: A comprehensive review. Aquac. Fish..

[22] Wanzenböck J. (2021). Rearing and stocking of coregonids: A comparison of aquaculture practices in Eurasia and North America. Adv. Limnol..

[23] Bolger A.M., Lohse M., Usadel B. (2014). Trimmomatic: A flexible trimmer for Illumina sequence data. Bioinformatics.

[24] Haas B., Papanicolaou A., Yassour M., Grabherr M., Blood P.D., Bowden J., Couger M.B., Eccles D., Li B., Lieber M. (2013). *De novo* transcript sequence reconstruction from RNA-seq using the Trinity platform for reference generation and analysis. Nat. Protoc..

[25] Manni M., Berkeley M.R., Seppey M., Zdobnov E.M. (2021). BUSCO: Assessing genomic data quality and beyond. Curr. Protoc..

[26] Haas B.J.T.D. TransDecoder Source. https://github.com/TransDecoder/TransDecoder.

[27] Buchfink B., Reuter K., Drost H.G. (2021). Sensitive protein alignments at tree-of-life scale using DIAMOND. Nat. Methods.

[28] Huerta-Cepas J., Szklarczyk D., Heller D., Hernández-Plaza A., Forslund S.K., Cook H., Mende D.R., Letunic I., Rattei T., Jensen L.J. (2019). eggNOG 5.0: A hierarchical, functionally and phylogenetically annotated orthology resource based on 5090 organisms and 2502 viruses. Nucleic Acids Res..

[29] Chen Y., Lun A.T.L., Smyth G.K. (2016). From reads to genes to pathways: Differential expression analysis of RNA-Seq experiments using Rsubread and the edgeR quasi-likelihood pipeline. F1000Research.

[30] Langmead B., Salzberg S.L. (2012). Fast gapped-read alignment with Bowtie 2. Nat. Methods.

[31] Robinson M.D., McCarthy D.J., Smyth G.K. (2010). edgeR: A Bioconductor package for differential expression analysis of digital gene expression data. Bioinformatics.

[32] Gibbons F.D., Roth F. (2002). Judging the quality of gene expression-based clustering methods using gene annotation. Genome Res..

[33] Bu D., Luo H., Huo P., Wang Z., Zhang S., He Z., Wu Y., Zhao L., Liu J., Guo J. (2021). KOBAS-i: Intelligent prioritization and exploratory visualization of biological functions for gene enrichment analysis. Nucleic Acids Res..

[34] Stefanovic D.I., Manzon L.A., McDougall C.S., Boreham D.R., Somers C.M., Wilson J.Y., Manzon R.G. (2016). Thermal stress and the heat shock response in embryonic and young of the year juvenile lake whitefish. Comp. Biochem. Physiol. A Mol. Integr. Physiol..

[35] Bustin S.A., Benes V., Garson J.A., Hellemans J., Huggett J., Kubista M., Mueller R., Nolan T., Pfaffl M.W., Shipley G.L. (2009). The MIQE Guidelines: Minimum Information for Publication of Quantitative Real-Time PCR Experiments. Clin. Chem..

[36] Sun J., Liu Z., Quan J., Li L., Zhao G., Lu J. (2022). RNA-Seq analysis reveals alternative splicing under heat stress in rainbow trout (*Oncorhynchus mykiss*). Mar. Biotechnol..

[37] Shi K.-P., Dong S.-L., Zhou Y.-G., Li Y., Gao Q.-F., Sun D.-J. (2019). RNA-Seq reveals temporal differences in the transcriptome response to acute heat stress in the atlantic salmon (*Salmo salar*). Comp. Biochem. Physiol. Part D Genom. Proteom..

[38] Jong W.W., Caspers G.J., Leunissen J.A. (1998). Genealogy of the alpha-crystallin-small heat-shock protein superfamily. Int. J. Biol. Macromol..

[39] Tedesco B., Cristofani R., Ferrari V., Cozzi M., Rusmini P., Casarotto E., Chierichetti M., Mina F., Galbiati M., Piccolella M. (2022). Insights on Human Small Heat Shock Proteins and Their Alterations in Diseases. Front. Mol. Biosci..

[40] Jena K.K., Kolapalli S.P., Mehto S., Nath P., Das B., Sahoo P.K., Ahad A., Syed G.H., Raghav S.K., Senapati S. (2018). TRIM16 controls assembly and degradation of protein aggregates by modulating the p62-NRF2 axis and autophagy. EMBO J..

[41] Prajapati P., Gohel D., Shinde A., Roy M., Singh K., Singh R. (2020). TRIM32 regulates mitochondrial mediated ROS levels and sensitizes the oxidative stress induced cell death. Cell Signal..

[42] Jiang J., Shi Y., Shan Z., Yang L., Wang X., Shi L. (2012). Bioaccumulation, Oxidative stress and HSP70 expression in *Cyprinus carpio* L. exposed to microcystin-LR under laboratory conditions. Comp. Biochem. Physiol. Part C Toxicol. Pharmacol..

[43] Chen B., Feder M.E., Kang L. (2018). Evolution of heat-shock protein expression underlying adaptive responses to environmental stress. Mol. Ecol..

[44] Kelly N.I., Wilson C.C., Currie S., Burness G. (2018). Acclimation capacity of the cardiac HSP70 and HSP90 response to thermal stress in lake trout (*Salvelinus namaycush*), a stenothermal ice-age relict. Comp. Biochem. Physiol. B Biochem. Mol. Biol..

[45] Qiu X.-B., Shao Y.-M., Miao S., Wang L. (2006). The Diversity of the DnaJ/Hsp40 family, the crucial partners for Hsp70 chaperones. Cell. Mol. Life Sci..

[46] Gabai V.L., Sherman M.Y. (2002). Invited review: Interplay between molecular chaperones and signaling pathways in survival of heat shock. J. Appl. Physiol..

[47] Melkani G.C., Cammarato A., Bernstein S.I. (2006). alphaB-crystallin maintains skeletal muscle myosin enzymatic activity and prevents its aggregation under heat-shock stress. J. Mol. Biol..

[48] Takagi Y., Yasuhara T., Gomi K. (2001). Creatine kinase and its isozymes. Rinsho Byori..

[49] Keceli G., Gupta A., Sourdon J., Gabr R., Schär M., Dey S., Tocchetti C.G., Stuber A., Agrimi J., Zhang Y. (2022). Mitochondrial creatine kinase attenuates pathologic remodeling in heart failure. Circ. Res..

[50] Moghadam-Kia S., Oddis C.V., Aggarwal R. (2016). Approach to asymptomatic creatine kinase elevation. Cleve Clin. J. Med..

[51] Morandi L., Angelini C., Prelle A., Pini A., Grassi B., Bernardi G., Politano L., Bruno C., De Grandis D., Cudia P. (2006). High plasma creatine kinase: Review of the literature and proposal for a diagnostic algorithm. Neurol. Sci..

[52] Li R., Wang B., Wu C., Li D., Wu Y., Ye L., Ye L., Chen X., Li P., Yuan Y. (2021). Acidic fibroblast growth factor attenuates type 2 diabetes-induced demyelination via suppressing oxidative stress damage. Cell Death Dis..

[53] Suomalainen A. (2013). Fibroblast growth factor 21: A novel biomarker for human muscle-manifesting mitochondrial disorders. Expert. Opin. Med. Diagn..

[54] Farooq M., Khan A.W., Kim M.S., Choi S. (2021). The role of fibroblast growth factor (FGF) signaling in tissue repair and regeneration. Cells.

[55] Cui Y., Hao Y., Li J., Bao W., Li G., Gao Y., Gu X. (2016). Chronic heat stress induces immune response, oxidative stress response, and apoptosis of finishing pig liver: A proteomic approach. Int. J. Mol. Sci..

[56] Merk M., Mitchell R.A., Endres S., Bucala R. (2012). D-dopachrome tautomerase (D-DT or MIF-2): Doubling the MIF cytokine family. Cytokine.

[57] Dimauro I., Antonioni A., Mercatelli N., Caporossi D. (2018). The role of αB-crystallin in skeletal and cardiac muscle tissues. Cell Stress Chaperones.

[58] Rothbard J.B., Kurnellas M.P., Brownell S., Adams C.M., Su L., Axtell R.C., Chen R., Fathman C.G., Robinson W.H., Steinman L. (2012). Therapeutic effects of systemic administration of chaperone αB-crystallin associated with binding proinflammatory plasma proteins. J. Biol. Chem..

[59] D’Amico D., Fiore R., Caporossi D., Di Felice V.D., Cappello F., Dimauro I., Barone R. (2021). Function and fiber-type specific distribution of Hsp60 and αB-Crystallin in skeletal muscles: Role of physical exercise. Biology.

[60] Gangalum R.K., Atanasov I.C., Zhou Z.H., Bhat S.P. (2011). αB-crystallin is found in detergent-resistant membrane microdomains and is secreted via exosomes from human retinal pigment epithelial cells. J. Biol. Chem..

[61] Boshans R.L., Szanto S., van Aelst L., D’Souza-Schorey C. (2000). ADP-ribosylation factor 6 regulates actin cytoskeleton remodeling in coordination with Rac1 and RhoA. Mol. Cell. Biol..

[62] Wang K., Wang S., Wang T., Xia Q., Xia S. (2024). The *Sclerotinia sclerotiorum* ADP-ribosylation factor 6 plays an essential role in abiotic stress response and fungal virulence to host plants. J. Fungi.

[63] Mori M., Shibasaki Y., Namba A., Yabu T., Wada N., Shiba H., Anzai H., Mano N. (2022). Alteration of hemoglobin ß gene expression in mucosal tissues of Japanese flounder, *Paralichthys olivaceus*, in response to heat stress, *Edwardsiella piscicida* infection, and immunostimulants administration. Fish Shellfish Immunol. Rep..

[64] Giordano D., Corti P., Coppola D., Altomonte G., Xue J., Russo R., di Prisco G., Verde C. (2021). Regulation of globin expression in Antarctic fish under thermal and hypoxic stress. Mar. Genom..

[65] Goldspink G. (1995). Adaptation of fish to different environmental temperature by qualitative and quantitative changes in gene expression. J. Therm. Biol..

[66] Agarwal M., Sharma A., Kumar P., Kumar A., Bharadwaj A., Saini M., Kardon G., Mathew S.J. (2020). Myosin heavy chain-embryonic regulates skeletal muscle differentiation during mammalian development. Development.

[67] Steinbacher P., Wanzenböck J., Brandauer M., Holper R., Landertshammer J., Mayr M., Platzl C., Stoiber W. (2017). Thermal experience during embryogenesis contributes to the induction of dwarfism in whitefish *Coregonus lavaretus*. PLoS ONE.

[68] Rasmussen M., Feng H.Z., Jin J.P. (2022). Evolution of the N-terminal regulation of cardiac troponin I for heart function of tetrapods: Lungfish presents an example of the emergence of novel submolecular structure to lead the capacity of adaptation. J. Mol. Evol..

[69] Wakabayashi T. (2015). Mechanism of the calcium-regulation of muscle contraction in pursuit of its structural basis. Proc. Jpn. Acad. Ser. B Phys. Biol. Sci..

[70] Santana Evangelista K., Andrich F., Figueiredo de Rezende F., Niland S., Cordeiro M.N., Horlacher T., Castelli R., Schmidt-Hederich A., Seeberger P.H., Sanchez E.F. (2009). Plumieribetin, a fish lectin homologous to mannose-binding B-type lectins, inhibits the collagen-binding alpha1beta1 integrin. J. Biol. Chem..

[71] Russell S., Lumsden J.S. (2005). Function and heterogeneity of fish lectins. Vet. Immun. Immunopath..

[72] Tsutsui S., Okamoto M., Tsasumi S., Suetake H., Kikuchi K., Suzuki Y. (2006). Novel mannose-specific lectins found in torafugu, Takifugu rubripes: A review. Comp. Biochem. Physiol..

[73] Zhang H., Zhao M., Liu Y., Zhou Z., Guo J. (2018). Identification of cytochrome P450 monooxygenase genes and their expression in response to high temperature in the alligatorweed flea beetle *Agasicles hygrophila* (Coleoptera: Chrysomelidae). Sci. Rep..

[74] Wang Y.C., Chang Y.W., Bai J., Zhang X.X., Iqbal J., Lu M.X., Hu J., Du Y.Z. (2021). High temperature stress induces expression of CYP450 genes and contributes to insecticide tolerance in *Liriomyza trifolii*. Pestic. Biochem. Physiol..

[75] Uno T., Ishizuka M., Itakura T. (2012). Cytochrome P450 (CYP) in fish. Environ. Toxicol. Pharmacol..

[76] Rendic S.P., Peter Guengerich F. (2018). Human cytochrome P450 enzymes 5-51 as targets of drugs and natural and environmental compounds: Mechanisms, induction, and inhibition-toxic effects and benefits. Drug Metab. Rev..

[77] Meer D.L., Marques I.J., Leito J.T., Besser J., Bakkers J., Schoonheere E., Bagowski C.P. (2006). Zebrafish cypher is important for somite formation and heart development. Dev. Biol..

[78] Zhou Q., Chu P.H., Huang C., Cheng C.F., Martone M.E., Knoll G., Shelton G.D., Evans S., Chen J. (2001). Ablation of cypher, a PDZ-LIM domain Z-line protein, causes a severe form of congenital myopathy. J. Cell Biol..

[79] Blech-Hermoni Y., Subedi K., Silver M., Jensen L., Coscia S., Kates M.M., Zhao Y., Raley C., Edwards N., Tran B. (2023). Expression of LIM domain-binding 3 (LDB3), a striated muscle Z-band alternatively spliced PDZ-motif protein in the nervous system. Sci. Rep..

[80] Dagoudo M., Mutebi E.T., Qiang J., Tao Y.F., Zhu H.J., Ngoepe T.K., Xu P. (2023). Effects of acute heat stress on haemato-biochemical parameters, oxidative resistance ability, and immune responses of hybrid yellow catfish (*Pelteobagrus fulvidraco* × *P. vachelli*) juveniles. Vet. Res. Commun..

[81] Tang S., Liang J., Xiang C., Xiao Y., Wang X., Wu J., Li G., Cheke R.A. (2019). A general model of hormesis in biological systems and its application to pest management. J. R. Soc. Interface.

[82] Nandi S.K., Panda A.K., Chakraborty A., Rathee S., Roy I., Barik S., Mohapatra S.S., Biswas A. (2022). Role of ATP-small heat shock protein interaction in human diseases. Front. Mol. Biosci..

[83] Khan M.N., Siddiqui M.H., AlSolami M.A., Siddiqui Z.H. (2024). Melatonin-regulated heat shock proteins and mitochondrial ATP synthase induce drought tolerance through sustaining ROS homeostasis in H2S-dependent manner. Plant Physiol. Biochem..

[84] Johnston I.A., McLay H.A., Abercromby M., Robins D. (2000). Early thermal experience has different effects on growth and muscle fibre recruitment in spring- and autumn-running Atlantic salmon populations. J. Exp. Biol..

[85] Johnston I.A., Abercromby M., Vieira V.L.A., Sigursteindóttir R.J., Kristjánsson B.K., Sibthorpe D., Skúlason S. (2004). Rapid evolution of muscle fibre number in post-glacial populations of arctic charr *Salvelinus alpinus*. J. Exp. Biol..

[86] Hu C., Yang J., Qi Z., Wu H., Wang B., Zou F., Mei H., Liu J., Wang W., Liu Q. (2022). Heat shock proteins: Biological functions, pathological roles, and therapeutic opportunities. MedComm.

[87] Campos C., Valente L.M., Conceição L.E., Engrola S., Fernandes J.M. (2013). Temperature affects methylation of the myogenin putative promoter, its expression and muscle cellularity in Senegalese sole larvae. Epigenetics.

[88] Lopez-Maury L., Marguerat S., Bahler J. (2008). Tuning gene expression to changing environments: From rapid responses to evolutionary adaptation. Nat. Rev. Genet..

[89] Polato N.R., Altman N.S., Baums I.B. (2013). Variation in the transcriptional response of threatened coral larvae to elevated temperatures. Mol. Ecol..

[90] Koroleva A.G., Vakhteeva E.A., Epifantsev A.A., Sukhanova L.V., Yakhnenko V.M., Glyzina O.Y., Tolstikova L.I., Cherezova V.M., Sidorova T.V., Potapov S.A. (2024). Acclimation during Embryogenesis Remodulates Telomerase Activity and Gene Expression in Baikal Whitefish Larvae, Mitigating the Effects of Acute Temperature Stress. Animals.

[91] Heikkila J.J. (2017). The expression and function of hsp30-like small heat shock protein genes in amphibians, birds, fish, and reptiles. Comp. Biochem. Physiol. A Mol. Integr. Physiol..

[92] Liu X., Shi H., Liu Z., Kang Y., Wang J., Huang J. (2019). Effect of Heat Stress on Heat Shock Protein 30 (Hsp30) mRNA Expression in Rainbow Trout (*Oncorhynchus mykiss*). Turk. J. Fish. Aquat. Sci..

[93] Muchowski P.J., Schaffar G., Sittler A., Wanker E.E., Hayer-Hartl M.K., Hartl F.U. (2000). Hsp70 and hsp40 chaperones can inhibit self-assembly of polyglutamine proteins into amyloid-like fibrils. Proc. Natl. Acad. Sci. USA.

[94] Ishida Y., Nagata K. (2011). Hsp47 as a collagen-specific molecular chaperone. Methods Enzymol..

