# Supplementary material for: Transcriptional Rearrangements Associated with Thermal Stress and Preadaptation in Baikal Whitefish (Coregonus baicalensis)
Source: Animals (Basel). 2024 Oct 25;14(21):3077. doi: 10.3390/ani14213077 (PMC11545380; doi:10.3390/ani14213077)
Supplement: Supplementary file 1 [file animals-14-03077-s001.zip › S1_Transcriptome assembly_report.pdf]

Report

	trinity_out_dir.Trinity
# contigs (>= 0 bp)	727333
# contigs (>= 1000 bp)	120898
# contigs (>= 5000 bp)	2984
# contigs (>= 10000 bp)	104
# contigs (>= 25000 bp)	0
# contigs (>= 50000 bp)	0
Total length (>= 0 bp)	475538512
Total length (>= 1000 bp)	247858225
Total length (>= 5000 bp)	18880014
Total length (>= 10000 bp)	1258303
Total length (>= 25000 bp)	0
Total length (>= 50000 bp)	0
# contigs	236733
Largest contig	16494
Total length	328106313
GC (%)	47.67
N50	1762
N90	663
auN	2175.7
L50	57902
L90	179370
# N's per 100 kbp	0.00

All statistics are based on contigs of size >= 500 bp, unless otherwise noted (e.g., "# contigs (>= 0 bp)" and "Total length (>= 0 bp)" include all contigs).

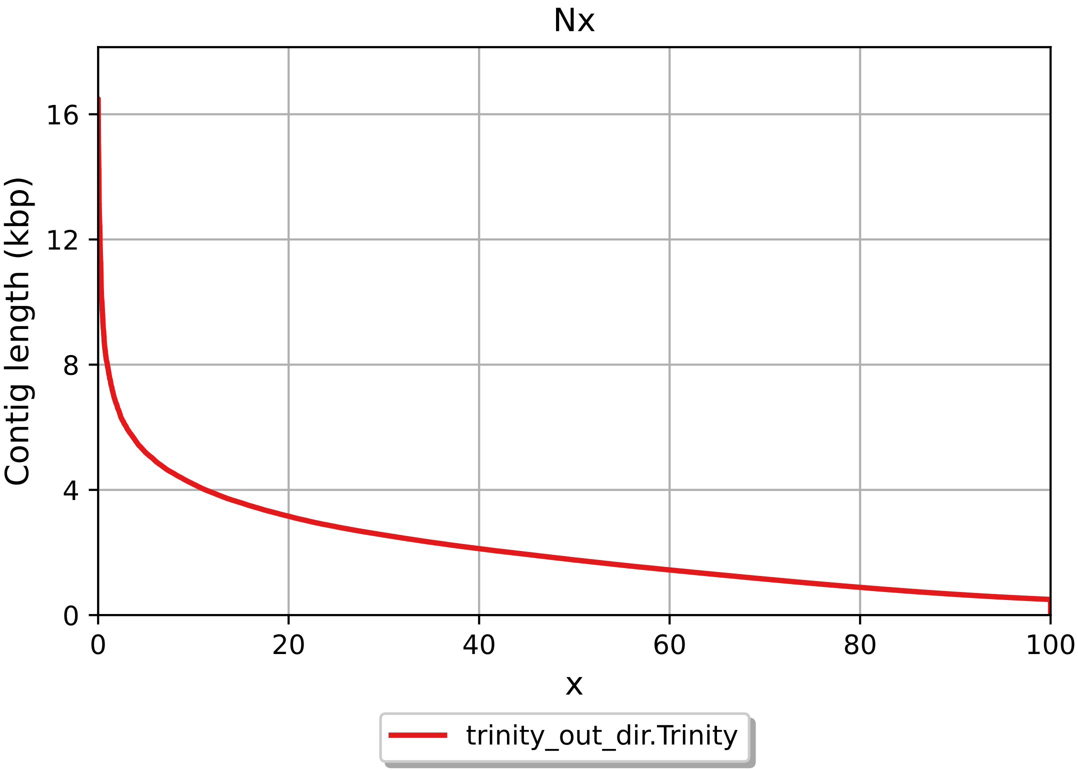

Nx
Contig length (kbp)
0
4
8
12
16
0
20
40
60
80
100
x
trinity_out_dir.Trinity

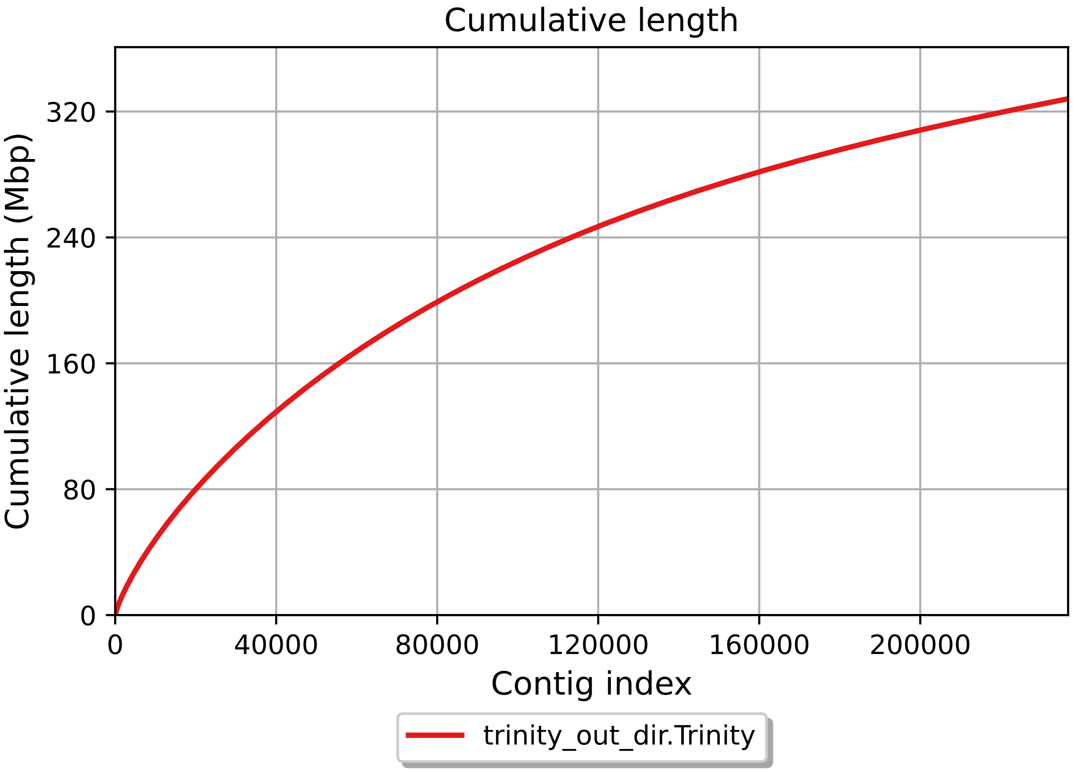
Cumulative length
Cumulative length (Mbp)
0
80
160
240
320
0
40000
80000
120000
160000
200000
Contig index
trinity_out_dir.Trinity

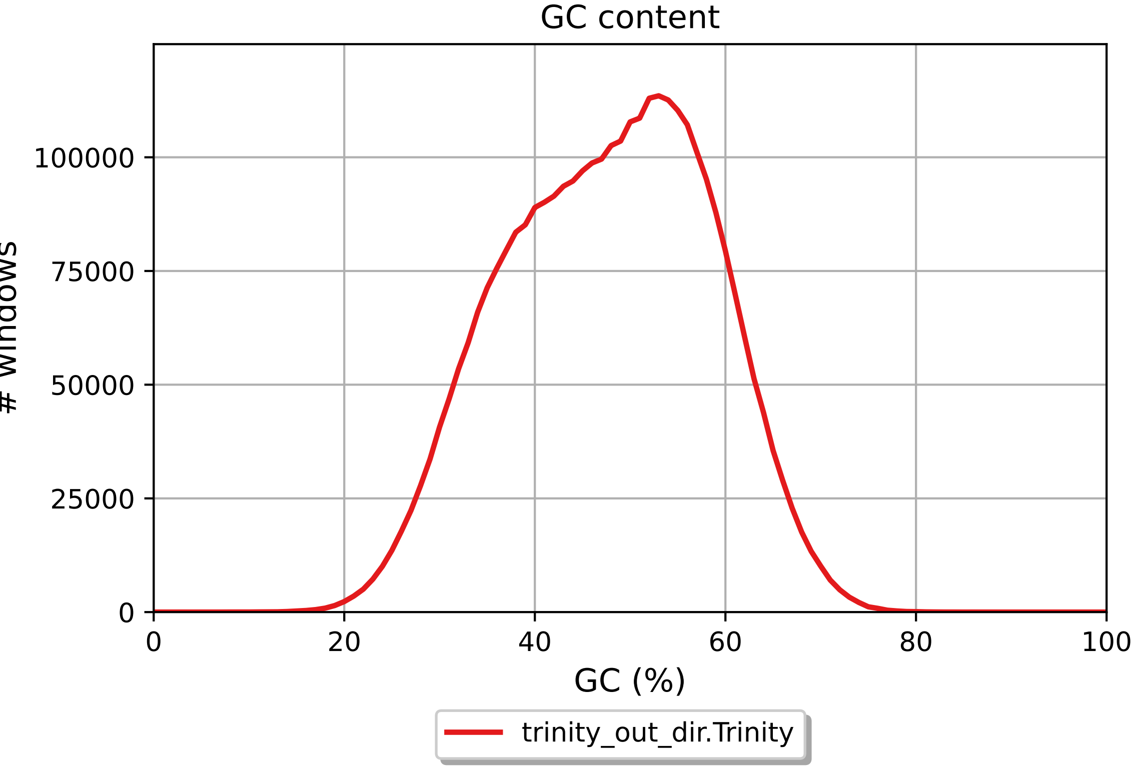
GC content
100000
75000
50000
25000
0
windows
0
20
40
60
80
100
GC (%)
trinity_out_dir.Trinity

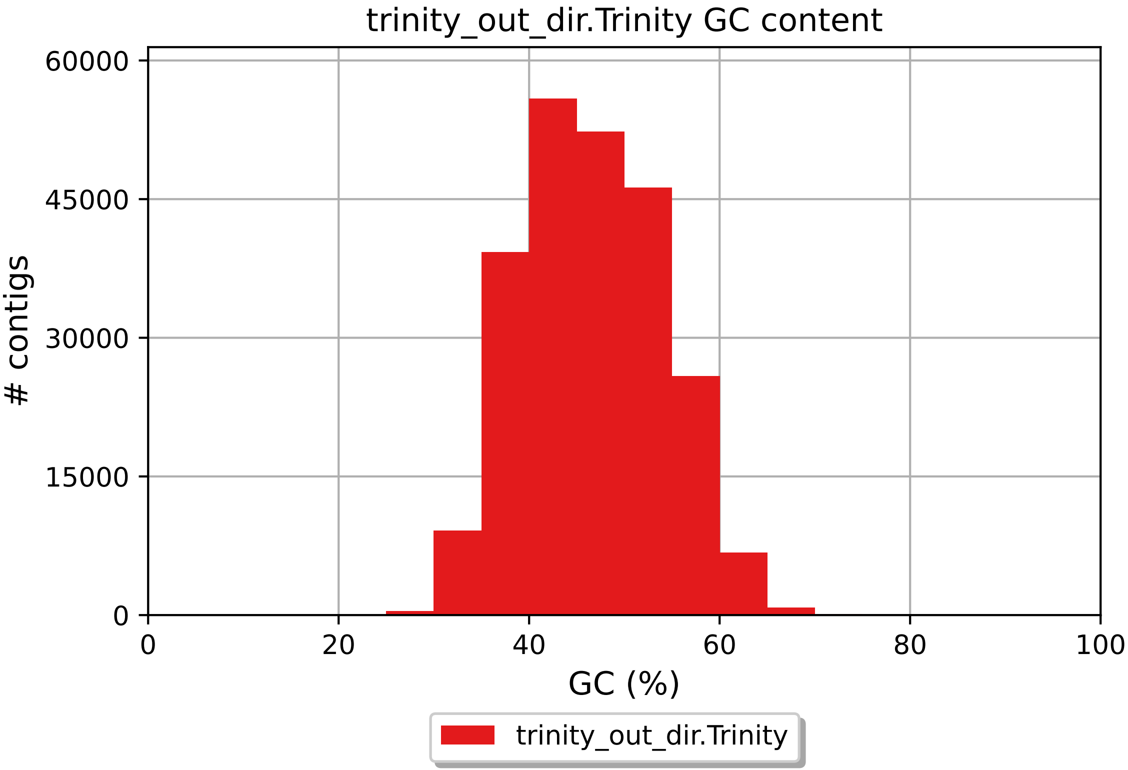
trinity_out_dir.Trinity GC content
60000
45000
30000
15000
0
contigs
0
20
40
60
80
100
GC (%)
trinity_out_dir.Trinity
